# Who will step up? A cross-sectional study on factors associated with medical students’ willingness to provide abortion care

**DOI:** 10.1371/journal.pone.0362064

**Published:** 2026-11-06

**Authors:** Mirja Baumgart, Anette Rohmann, Mareike Rutenkröger, Jördis Maria Zill, Anja Lindig

**Affiliations:** 1 Department of Medical Psychology, University Medical Center Hamburg-Eppendorf, Hamburg, Germany; 2 Faculty of Psychology, FernUniversität in Hagen, Hagen, Germany; 3 Department of Psychology, Faculty of Medicine and Health Sciences, University Medicine Oldenburg, Carl von Ossietzky Universität Oldenburg, Oldenburg, Germany; University of Foggia: Universita degli Studi di Foggia, ITALY

## Abstract

**Introduction:**

The decreasing number of physicians performing abortions in Germany limits access to high-quality care for individuals with unintended pregnancy seeking abortions. To address the barriers preventing higher participation in abortion care, the aim of this study was to quantitatively explore the attitudes of medical students towards abortion. Furthermore, factors associated with medical students’ attitudes towards abortion and their willingness to participate in abortion care were examined based on intergroup contact theory and the theory of planned behaviour.

**Methods:**

A Germany-wide online survey was conducted. Validated scales and single items were translated into German using the TRAPD protocol and adapted for medical students via cognitive interviews (*n* = 10). Survey data from *n* = 233 medical students were analysed using descriptive statistics, regression, and mediation analyses with abortion attitudes and willingness to provide abortion care as criteria.

**Results:**

Participants had overall positive attitudes towards abortion. Female gender and low religiosity were associated with positive attitudes. Fear of stigmatisation and perceived knowledge were negatively associated with attitudes towards abortion. Positive attitudes towards abortion were significantly associated with higher willingness to participate in abortion care. The association between attitudes and contact, as well as the expected mediation effects of fear of stigmatisation, perceived knowledge and perspective-taking, were nonsignificant.

**Conclusions:**

This study demonstrates the association of medical students’ attitudes towards abortion with their willingness to participate in abortion care in the future. However, lack of perceived knowledge and fear of stigmatisation may represent barriers for young physicians to provide abortion care in their later work life. Addressing structural barriers with targeted interventions and training programmes is essential that young physicians feel equipped, supported and safe to provide abortion care.

**Trial registration:**

The study was registered via AsPredicted.org prior to data collection (September 8^th^, 2023). Trial registration number: 142964.

## Introduction

Abortion, though a common gynaecological procedure, is illegal according to the German Criminal Code (§218 ff. StGB), but remains non-punishable in case of a medical indication (§218a, paragraph 2 StGB), criminological indication (§218a, paragraph 3 StGB) or according to consultation regulation (§218a, paragraph 1 StGB). The latter includes abortion performed by a physician within the first 12 weeks of pregnancy after conception, following mandatory counselling and a three-day waiting period (§218a StGB). In recent years, abortion law has been a politically contentious issue in Germany. In 2022, the prohibition on physicians offering abortion-related information was lifted (§219a StGB). In 2024, an expert panel, appointed by the German federal government, recommended legalising first-trimester abortions [1]. By 2027, abortion should be mandatory in the medical school curriculum [2]. Internationally, recent years have seen shifts in abortion legislation, including both liberalisation and restriction. In 2022, the U.S. Supreme Court overturned Roe v. Wade, which had protected the right to legal abortion since 1973 [3]. Shortly after taking office, new Republican President Donald Trump imposed policies restricting abortion care in the U.S., signalling further limitations ahead [4]. Similarly, Poland implemented a near-total ban on abortion during the COVID-19 pandemic, further tightening its already restrictive legislation [5]. Germany is also witnessing some political movements advocating for stricter access to abortion [6]. A recent attempt to decriminalise abortion before the early federal elections and the end of the legislative period has failed [7]. However, previous research suggests that restricting abortion access does not necessarily reduce the number of abortions but rather forces individuals to seek unsafe procedures, thereby endangering their health and lives [8,9]. In 2020, World Health Organization has therefore included comprehensive abortion care in the list of essential health care services [9].

However, access to abortion care in Germany remains restricted: Abortion is still stigmatised, with many physicians choosing not to offer the procedure, often due to religious objections [10]. Between 2003 and 2021, the number of clinics and practices offering abortion care in Germany (“Meldestellen”) decreased from 2 050 to 1 092 [11]. Regional disparities further exacerbate the deficient access to abortion care, with northern and eastern Germany offering a higher density of providers than the southern and western regions [12]. Access remains particularly limited in rural areas [12]. Moreover, physicians are legally permitted to refuse to perform abortions based on personal convictions (§12 SchKG). Due to those limits in access to abortion care, individuals – particularly those from rural areas – often have to travel significant distances to access abortion care [12,13]. A recent qualitative study by Lindig et al. (2024) involving 18 experts in psycho-social or medical abortion care emphasised that the lack of abortion care providers significantly limits person-centred abortion care [13].

In Germany, the average age of gynaecologists is 54 years, with 30% aged 60 or older [14]. Thus, a substantial proportion of providers are nearing retirement. As a result, access to abortion services may become increasingly limited in the future. Consequently, it is of great societal importance to train future medical professionals with the necessary skills to perform abortions and address gaps in healthcare provision. However, in most medical schools, abortion is not yet part of the curriculum, and it is not a mandatory component of gynaecology training, limiting student exposure to abortion care [10]. Due to the stigmatisation of individuals seeking or performing abortions, future physicians may feel apprehensive about the topic and hesitant to engage with individuals seeking abortion services [15,16]. Intergroup contact theory however suggests that positive interactions with stigmatised groups can foster positive attitudes, mediated by an increase of knowledge about the stigmatised group, a decrease of anxiety, and an increase in empathy and perspective-taking [17,18]. Studies on abortion education have shown that contact with individuals seeking abortions during internships improved medical students’ knowledge, empathy, and willingness to provide abortion care [19,20]. According to the theory of planned behaviour, attitudes as well as social norms and perceived behavioural control influence intentions to perform a certain behaviour, which is a strong predictor of behaviour itself [21]. International studies showed that physicians with more positive attitudes towards abortion are more likely to provide abortions [22,23]. Moreover, research on the general public's attitudes towards abortion has indicated that demographic factors, including age, gender, place of residence and religiosity, have a significant impact on attitudes towards abortion [24,25].

Despite the ongoing discourse surrounding abortion law and education, to date only one quantitative and few qualitative studies have explored the attitudes of medical students and physicians towards abortion in Germany [10,13,26]. In addition to the above-mentioned study by Lindig et al. [13], Baier and Behnke [10] conducted an interview study with 14 medical students and four physicians and revealed barriers for specialisation and participation in abortion care including misinformation, lack of knowledge, fear of stigmatisation, and religious beliefs. In a cross-sectional study drawing on the theory of planned behaviour by Le et al. with 213 gynaecologists and abortion providers of all specialties, perceived behavioural control was the strongest predictor of intention to provide abortion care, followed by subjective norms and attitudes towards abortion. Abortion providers reported significantly more positive attitudes, stronger subjective norms, higher perceived behavioural control, lower fear of stigmatisation and higher subjective knowledge of abortion care in comparison to non-providers [26].

Based on those findings, intergroup contact theory [17,18], and the theory of planned behaviour [21], the aim of the present study was threefold: 1) to quantitatively assess medical students’ abortion attitudes, 2) to investigate whether demographic factors (including age, gender, religiosity and state of origin), contact with individuals seeking abortions, perceived knowledge, fear of stigmatisation, and perspective-taking are associated with these attitudes, and 3) to examine whether medical students’ attitude towards abortion, subjective norms, and perceived behavioural control are associated with their willingness to provide abortion care.

## Materials and methods

### Study design

This quantitative cross-sectional study assessed attitudes towards abortion and associated factors in a sample of medical students in Germany via an online survey. The inclusion criterion for the survey was a current enrolment in a medical school in Germany. The study was preregistered on AsPredicted (registration number 142964, https://aspredicted.org/b5b2q.pdf). For reporting of results, the STROBE cross sectional reporting guidelines were used (see S1 Appendix) [27].

### Ethics approval and consent to participate

This study was carried out according to the latest version of the Helsinki Declaration of the World Medical Association, respected principles of good scientific practice and met standards of research ethics. The study was approved by the Local Ethics Committee of the University Medical Center Hamburg-Eppendorf, Germany (LPEK-0628, May 27^th^, 2023). Data protection and confidentiality requirements were met. Study participation was voluntary. All participants received written information about the aims of the study, data collection, and the use of collected data and gave informed consent before participation.

### Measures

A survey was developed using validated scales, items from similar international studies, and self-developed items. Whenever possible, validated and up-to-date scales previously tested on the target population and available in a validated German translation were used. Consequently, for the measurement of the constructs abortion attitudes, perspective-taking, and fear of stigmatisation, validated scales were included: To assess attitudes towards abortion, the 14-item Abortion Attitude Scale (AAS) was used, originally developed in the U.S. in 1983 for classroom use with student populations [28]. Perspective-taking was measured using the German translation of the Jefferson Scale of Physician Empathy for Students (JSPE), validated in a sample of *n* = 516 Austrian medical students [29]. Ten items were selected that loaded onto the perspective-taking factor. Fear of stigmatisation was assessed using the 10-item Disclosure Management subscale of the Revised Abortion Providers Stigma Scale (APSS) [16]. To assess contact with individuals seeking abortions, perceived knowledge about abortion, subjective norms (operationalised as relevant others’ perceived attitudes towards abortion), perceived behavioural control, and willingness to participate in abortion care, single items from existing studies as well as self-developed items were used and adapted to the target audience. Sociodemographic data included items on age, gender, current semester in medical school, future preferred medical specialisation, religiosity, state of origin, and state of residence. To assess religiosity, two items on influence of faith and visits of religious sites were included.

The AAS and the APSS subscale, which were only available in English language, were translated and culturally adapted by the study team (AL, MB, MR) using the team translation protocol TRAPD (Translation, Review, Adjucation, Pre-testing, and Documentation) [30]. The AAS had to be adapted explicitly for the German cultural and political context of the present. Thus, after data collection for the study at hand, we conducted a secondary analysis to assess psychometric properties of the AAS using the data set collected in the present study with medical students [31]. In accordance with the COSMIN criteria (Consensus-based Standards for the Selection of Health Measurement Instruments) [32], three rounds of cognitive interviews with *n* = 10 medical students (age: *M* = 24.80 years, *SD* = 2.04; 7 female) from different medical schools in three German states were conducted to ensure comprehensibility and suitability for the study population, followed by discussions and adaptions by the study team (AL, MB, MR). The survey was again adapted and finalised after written feedback by *n* = 2 counsellors in social support services and *n* = 3 gynaecologists providing abortion care.

Table 1 provides an overview of the included instruments and items, along with item number, scale range, and example items. An attention check with two items was included to test for careful responses [33]. The survey further included various additional items on attitudes towards abortion, as well as single items on objective knowledge about abortion and other constructs. These data were not analysed within the scope of this study. For the complete survey including descriptive results of all analysed and additional items, please see S2 Appendix.

**Table 1:** Instruments and constructs used in the survey of n = 233 medical students in Germany.

Instrument/ Construct	Items/Scale	Source	Translation	Adaptation	Example Item
Abortion Attitude Scale (AAS)	14 items/6-point: “strongly disagree” to “strongly agree”	Sloan, 1983 [28]	Y	Y	Abortion is a good way of solving an unwanted pregnancy.
German Jefferson Scale of Physicians Empathy for Students (JSPE-S), Perspective-taking dimension	10 items/6-point: “strongly disagree” to “strongly agree”	Preusche & Wagner-Menghin, 2013 [29]	N	N	Physicians should try to stand in their patients’ shoes when providing care to them.
Revised Abortion Provider Stigma Scale (APSS), Subscale “disclosure management”	10 items/5-point: “never” to “all of the time”	Martin et al., 2018 [16]	Y	Y	I worry about telling people I work in abortion care.
Contact with individuals seeking abortions	3 items/5-point: “not at all” to “daily”	self-developed	n/a	n/a	How often do you have contact with people in your close circle of friends or family who have had an abortion?
Perceived knowledge regarding abortion care	4 items/5-point: “not at all” to “extremely”	Cohen et al., 2021 [42]; Pace et al., 2008 [19]	Y	Y	My medical school education on abortion covered everything I need to know.
Subjective norms	5 items/5-point: “oppose a lot” to “support a lot”	Schmuhl et al., 2021 [22]	Y	Y	Please indicate or estimate whether the following individuals oppose or support unrestricted access to abortion. – Family members.
Perceived behavioural control	3 items/5-point: “strongly disagree” to “strongly agree”	self-developed	n/a	n/a	Whether I perform abortions in my future career will depend solely on my own decision.
Willingness to participate in abortion care	4 items/4-point: “no, under no circumstances” to “yes, in any legally justifiable circumstances”	Cohen et al., 2021 [42]	Y	Y	In your future career as a doctor, would you be willing to be involved in the following aspects of abortion care (assuming that you had the relevant training and experience)? – Perform medical abortions.
Demographic items	n/a	self-developed; Schmuhl et al, 2021 [22]	n/a; Y	n/a; Y	Age, gender, preferred medical specialisation, religiosity (influence of faith, visits of religious sites), state of residence and origin

Y = yes, N = no, n/a = not applicable.

### Data collection

Data were collected between September 8^th^ and October 4^th^, 2023, using Unipark (EFS Survey, Tivian XI GmbH). The survey aimed to reach as many as possible of all 113.383 medical students enrolled in German universities with medical schools in 2023 [34]. For this purpose, these institutions were first identified, and a list of all 42 German medical schools (36 public, 6 private) at the time with medical student councils and their email addresses was compiled. Five medical schools either did not provide contact details (*n* = 4) or stated directly that they do not distribute survey links (*n* = 1) and were thus not contacted. Medical school student councils of 37 medical schools (35 public, 2 private) were contacted between September 8^th^ and 18^th^, 2023 via email and asked to distribute the survey link via mailing lists, social media and digital bulletin boards. A table showing the distribution of contacted medical schools per federal state can be found in S3 Appendix. However, in the absence of any feedback regarding the process of link distribution, it is not possible to draw any inferences regarding the number of students who were actually reached or the medical schools, which were represented in our sample. Participation was voluntary and without financial compensation. The survey did not include any mandatory items, so that participants could decide for each item, whether they want to answer it or not. Participants received study information when opening the online survey via the link and provided informed consent before proceeding by clicking a box after reading the study information.

### Data analysis

Only completed surveys were included in the analysis. Data sets from participants who discontinued the survey before the final question were excluded. Participants were also excluded if they indicated that they were not currently enrolled in a medical school in Germany and therefore did not meet the inclusion criterion, answered control items incorrectly, or had suspicious, i.e., careless or effortful, response patterns [33]. Suspicious response was identified based on completion time being more than two standard deviations below the average, repeated selection of the same response option, extreme response behaviour, or indications that the survey may have been completed multiple times by the same individual [33]. For multi-item scales, person-level mean imputation within a scale (i.e., replacing a missing value with the mean of the remaining items completed by the same participant) [35] was applied when no more than 10% of the items within a scale were missing. We chose this threshold a priori as a pragmatic criterion, ensuring that imputation was only performed when the proportion of missing item information was small. Thus, imputation was only applicable to scales comprising at least ten items. Therefore, this imputation was only applied to the scales assessing abortion attitudes (AAS, 14 items), fear of stigmatisation (APSS, 10 items) and perspective taking (JSPE, 10 items). For scales containing less than ten items or in case of missing single items, even a single missing item would exceed the predefined threshold and participants with missing item responses were excluded from the respective scale analysis. Preferred medical specialisation and state of residence and origin were dichotomised for analysis into “communicative specialisation” (e.g., general medicine) or “technical specialisation” (e.g., radiology) according to Hojat et al. [36] and “old” (e.g., Bavaria) or “new” federal states (i.e., the territory of the former German Democratic Republic, e.g., Saxony).

Instruments were tested for reliability using Cronbach’s α or Spearman-Brown coefficient (in case of 2-item-scales). Items were removed if necessary to achieve acceptable internal consistency (α ≥ .60) [37]. Requirements for multiple linear regression were tested, and if unmet, the bootstrap method with 5 000 repetitions was applied [38]. Effect sizes were interpreted following Cohen’s convention [39].

For the primary objectives 2) and 3), multiple linear regression analyses were conducted with attitudes towards abortion and willingness to participate in abortion care as criteria, and age, gender, religiosity and state of origin, and attitudes towards abortion, subjective norms and perceived behavioural control, respectively, as predictors. Statistically significant demographic factors were included in all further regression and mediation analyses. To further analyse the relationship between contact and attitudes as a secondary objective, parallel mediation analyses were performed with contact as predictor, attitudes towards abortion as criterion, and perceived knowledge, perspective-taking, and fear of stigmatisation as mediators, controlling for gender and religiosity. Data analysis was conducted using IBM SPSS Statistics (version 29.0.0.0).

### Sample size

An a priori power analysis for the primary objective using G*Power (Version 3.1.9.7) [40], based on a multiple regression analysis with one criterion (attitudes towards abortion) and nine predictors (age, gender, preferred medical specialisation, religiosity, state of origin, contact, perceived knowledge, fear of stigmatisation, and perspective-taking) with small to medium sized expected effects of f^2^ = .09, a power of  .80 and a level of significance of  .05, revealed a necessary sample size of 183 participants. This sample size was also deemed appropriate for the multiple regression analysis with willingness to participate in abortion care as criterion and eight predictors (attitudes towards abortion, subjective norms, perceived behavioural control as well as the potential control variables gender, preferred medical specialisation, religiosity, and state of origin). Adding an additional 20% to account for missing values and exclusions, a target sample size of 220 was determined for the primary objectives. For the secondary objective, the mediation analysis with three mediators, the calculated sample size was confirmed suitable via Monte-Carlo power analysis [41]. All calculations were made assuming small to medium sized effects between contact and prejudice, as well as knowledge, fear of stigmatisation and perspective-taking, based on the meta-analyses by Pettigrew and Tropp [17,18].

## Results

### Description of data sets and sample characteristics

Of the 351 participants who opened the survey link within the period of data collection, 233 participants (66.4%) were included in analyses. Fifteen participants (4.3%) did not answer a single question, 96 (27.4%) did not finish the survey. Of the 240 participants who completed the survey, three participants did not meet the inclusion criteria, and two failed the attention check question. All remaining participants reported having taken part in the survey seriously. The average completion time was 16.4 minutes (*SD* = 6.7). No participants completed the questionnaire in less than two standard deviations below the mean completion time. Following inspection for suspicious response patterns, two additional participants were excluded for providing identical successive answers on the AAS scale and exhibiting response times more than three standard deviations below the mean, classifying them as outliers. The analysed sample size varied from 226 to 233, depending on the number of items completed for each construct.

Participants’ average age was 23.7 years (*SD* = 3.8), most participants were female (*n* = 193, 82.8%), aimed to become gynaecologists (*n* = 36, 15.6%), and identified themselves as Christians (*n* = 112, 48.1%). However, 152 participants (65.2%) stated that they were not influenced by religious beliefs, and 94 (40.3%) never visited religious sites. Participants were in their 1^st^ to 14^th^ semester of medical school (*Mdn* = 7). Most participants lived in North Rhine-Westphalia (*n* = 136, 58.4%), and 115 (49.8%) grew up there. Only 10.0% of participants grew up in the new federal states. Detailed demographic data can be found in Table 2.

**Table 2:** Descriptive statistics on demographic data of medical students participating in the survey (n = 233).

Variable	*N*	%
Gender		
Female	193	82.8
Male	37	15.9
Diverse/non-binary	1	0.4
Not specified	2	0.9
Preferred medical specialisation ^a^		
Communicative	147	63.1
Technical	84	36.1
Missing	2	0.9
State of origin ^b^		
Old Federal States	208	89.3
New Federal States	23	10.0
Missing	2	0.9
Religion		
None	109	46.8
Roman Catholic	57	24.5
Protestant	55	23.6
Islamic	5	2.1
Jewish	1	0.4
Other	6	2.6
Influence of faith		
I have no religious beliefs	116	49.8
Not at all	36	15.5
Low	43	18.5
Somewhat	19	8.2
Rather strong	15	6.4
Very strong	4	1.7
Visits of religious sites		
Never	94	40.3
Once a year	61	26.2
Several times a year	59	25.3
Several times a month	7	3.0
Once a week or more often	11	4.7
Missing	1	0.4

^a^Communicative: Child and adolescent psychiatry and psychotherapy, Dermatology, General medicine, Gynaecology and obstetrics, Internal medicine, Neurology, Occupational medicine, Paediatrics and adolescent medicine, Physical and rehabilitative medicine, Psychiatry and psychotherapy, Psychosomatic medicine and psychotherapy, Public health care, Urology; Technical: Anatomy, Anaesthesiology, Biochemistry, Cardiology, Forensic medicine, Haematology, Human genetics, Hygiene and environmental medicine, Neurosurgery, Nuclear medicine, Oncology, Ophthalmology, Oral and maxillofacial surgery, Otolaryngology, Pathology, Pharmacology, Phoniatrics, Physiology, Radiology, Radiation therapy, Transfusion medicine.

^b^Old Federal States: Baden-Württemberg, Bavaria, Berlin, Bremen, Hamburg, Hesse, Lower Saxony, North Rhine-Westphalia, Rhineland-Palatinate, Saarland, Schleswig-Holstein; New Federal States: Brandenburg, Mecklenburg-West Pomerania, Saxony, Saxony-Anhalt, Thuringia.

### Medical students’ attitudes towards abortion and associated factors

#### Descriptive results.

The instruments used were reliable, with validated scales showing good to very good internal consistency. Self-developed and adapted constructs had acceptable to good reliability, except for perceived knowledge (see Table 3 for reliabilities and descriptive statistics of all measured constructs).

**Table 3:** Descriptive statistics and Cronbach’s alpha of the constructs in the survey.

Construct	*Min*	*Max*	*M*	*SD*	α ^a^
Attitudes towards abortion (AAS) ^b^	1.50	5.93	4.77	0.82	.923
Fear of stigmatisation (APSS)	1.00	5.00	2.24	0.82	.939
Perspective-taking (JSPE-S) ^b^	3.90	6.00	5.51	0.44	.828
Contact	1.00	4.50	1.71	0.82	.749
Perceived knowledge ^c, d^	1.00	4.00	2.18	0.81	.629
Subjective norms	1.60	5.00	3.78	0.65	.765
Perceived behavioural control	1.00	5.00	3.46	1.43	n/a ^e^
Willingness to participate in care ^d^	1.00	4.00	3.64	0.60	.836

*N* = 233, if not stated otherwise. Scale range = 1.00–5.00, if not stated otherwise. ^a^ If scales consisted of only two items, Spearman-Brown-Coefficient instead of Cronbach’s α was calculated. ^b^ Scale range = 1.00–6.00. ^c^ *n* = 232. ^d^ Scale range = 1.00–4.00. ^e^ not applicable – single item.

Overall, students showed positive attitudes towards abortion and high perspective-taking ability. The mean scores for contact, perceived knowledge about abortion as well as fear of stigmatisation were low, indicating limited contact with abortion-seeking individuals and low perceived abortion-related knowledge. Nevertheless, students reported little fear of stigmatisation in case of future abortion provision. Participants’ mean scores for subjective norms and perceived behavioural control were high. The mean scores for willingness to participate in abortion care were high, but decreased with more specific involvement in abortion care, from advising about abortion options to performing surgical abortions: The mean willingness to counsel patients on abortion was *M* = 3.91 (*SD* = 0.39) on a 4-point scale from 1 (no, under no circumstances) to 4 (yes, in any legally justifiable circumstances), with 93.6% expressing unrestricted willingness under legal provisions. For referrals to abortion providers, 87.6% indicated willingness (*M* = 3.81, *SD* = 0.59). Willingness to perform abortions was lower, with 70.4% of participants who are willing to perform medication abortion (*M* = 3.51, *SD* = 0.87) and 60.5% who are willing to perform surgical abortion (*M* = 3.32, *SD* = 0.98) in the future.

### Associations between demographic factors and abortion attitudes

Attitudes towards abortion were positively correlated with gender (*r* = .32, *p* < .001) and negatively with influence of faith (*r* = −.53, *p* < .001) and visits of religious sites (*r* = −.42, *p* < .001). Gender was also correlated with preferred medical specialisation (*r* = .26, *p* < .001), with women favouring communicative fields. Influence of faith and visits of religious sites showed the strongest correlation (*r* = .75, *p* < .001). Pearson correlations of all variables can be found in S3 Appendix. A multiple linear regression showed that the proposed model explained 34.4% of the variance in abortion attitudes, *F*(6, 219) = 20.69, *p* < .001, adjusted *R*² = .344. Gender showed a moderately positive association with abortion attitudes (*B* = 0.61, BCa 95% CI [0.28, 0.95], *p* < .001, β = .28), with women having more positive abortion attitudes compared to men. Influence of faith was negatively associated with attitudes (*B* = −0.28, BCa 95% CI [−0.37, −0.19], *p* < .001, β = −.47). The association of attitudes towards abortion with age (*B* = −0.01, BCa 95% CI [−0.03, 0.01], *p* = .370, β = −.04), state of origin (*B* = −0.07, BCa 95% CI [−0.27, 0.17], *p* = .491, β = −.02), visits of religious sites (*B* = −0.05, BCa 95% CI [−0.16, 0.07], *p* = .390, β = −.06) as well as preferred medical specialisation (*B* = −0.14, BCa 95% CI [−0.32, 0.04], *p* = .144, β = −.08) were not clearly distinct from zero. Gender and influence of faith, which showed both statistically significant associations with attitudes towards abortion, were included as covariates in subsequent regression and mediation analyses. Detailed results of all regression analyses are shown in Tables 4–6.

**Table 4:** Regression of sociodemographic variables on attitudes towards abortion.

Variable	*B*	*SE* ^a^	β	*p* ^a^	BCa 95% CI ^a^
(Constant)	5.26	0.36		< .001	[4.48, 5.94]
Age	−0.01	0.01	−.04	.370	[-0.03, 0.01]
Gender ^b^	0.61	0.17	.28	< .001	[0.28, 0.95]
Specialisation ^c^	−0.14	0.09	−.08	.144	[-0.32, 0.04]
Influence of faith	−0.28	0.04	−.47	< .001	[-0.37, -0.19]
Visits of religious sites	−0.05	0.06	−.06	.390	[-0.16, 0.07]
State of origin ^d^	−0.07	0.10	−.02	.491	[-0.27, 0.14]

*n* = 226. *B* = Unstandardised regression coefficient. *SE* = Standard Error. β = Standardised regression coefficient. CI = Confidence Interval.

^a^Bootstrap based on 5 000 samples. ^b^ 0 = male, 1 = female. ^c^ 0 = technical, 1 = communicative. ^d^ 0 = Old Federal States, 1 = New Federal States.

**Table 5:** Regression of contact, perceived knowledge, fear of stigmatisation and perspective-taking on attitudes towards abortion.

Variable	*B*	*SE* ^a^	β	*p*	95% CI ^a^
(Constant)	5.71	0.65		< .001	[4.36, 6.96]
Contact	0.09	0.05	.09	.059	[-0.003, 0.191]
Perceived knowledge	−0.15	0.05	−.15	.003	[-0.25, -0.06]
Fear of stigmatisation	−0.28	0.07	−.28	< .001	[-0.41, -0.14]
Perspective-taking	−0.01	0.10	−.00	.936	[-0.20, 0.19]
Gender ^b^	0.55	0.15	.25	< .001	[0.27, 0.84]
Influence of faith	−0.26	0.04	−.44	< .001	[-0.34, -0.18]

*n* = 229. *B* = Unstandardised regression coefficient. *SE* = Standard Error. β = Standardised regression coefficient. CI = Confidence Interval.

^a^Bootstrap based on 5 000 samples. ^b^ 0 = male, 1 = female.

**Table 6:** Regression of attitudes, subjective norms and behavioural control on the willingness to participate in abortion care.

Variable	*B*	*SE* ^a^	β	*p* ^a^	BCa 95% CI ^a^
(Constant)	0.85	0.38		.024	[0.16, 1.72]
Attitudes towards abortion	0.50	0.05	.68	< .001	[0.39, 0.59]
Subjective norms	0.10	0.05	.11	.038	[0.01, 0.20]
Perceived behavioural control	−0.05	0.02	−.11	.008	[-0.08, -0.01]
Gender ^b^	0.11	0.07	.07	.124	[-0.03, 0.26]
Influence of faith	0.03	0.03	.06	.357	[-0.03, 0.08]

*n* = 228. *B* = Unstandardised regression coefficient. *SE* = Standard Error. β = Standardised regression coefficient. CI = Confidence Interval.

^a^Bootstrap based on 5 000 samples. ^b^ 0 = male, 1 = female.

### Intergroup effects on attitudes towards abortion

Attitudes towards abortion were positively correlated with contact with individuals seeking abortions (*r* = .13, *p* = .024) but negatively to perceived knowledge (*r* = −.14, *p* = .018) and fear of stigmatisation (*r* = −.39, *p* < .001). Fear of stigmatisation correlated positively with influence of faith (*r* = .24, *p* < .001), while perspective-taking showed a weak negative correlation with perceived knowledge (*r* = −.14, *p* = .019).

The tested mediation model with contact as predictor, attitudes towards abortion as criterion, and perceived knowledge, perspective-taking, and fear of stigmatisation as mediators explained 43.6% of the variance in abortion attitudes, *F*(6, 222) = 20.38, *p* < .001, adjusted *R*² = .436. The association between contact with individuals seeking abortions and attitudes towards abortion was very small (*B* = 0.09, BCa 95% CI [−0.003, 0.191], *p* = .059, β = .09). Perceived knowledge (*B* = −0.15, BCa 95% CI [−0.25, −0.06], *p* = .003, β = −.15) as well as fear of stigmatisation (*B* = −0.28, BCa 95% CI [−0.41, −0.14], *p* < .001, β = −.28) were negatively associated with attitudes towards abortion. Perspective-taking was not statistically associated with attitudes (*B* = −0.01, BCa 95% CI [−0.20, 0.19], *p* = .936, β = −.00). The mediation effect did not differ distinctly from zero: contact was not associated with perceived knowledge (*B* = 0.07, 95% CI [−0.07, 0.21], *p* = .336), fear of stigmatisation (*B* = −0.01, 95% CI [−0.14, 0.12], *p* = .834), or perspective-taking (*B* = −0.04, 95% CI [−0.13, 0.04], *p* = .314).

### Factors associated with willingness to participate in abortion care

Willingness to participate in abortion care was at least moderately correlated with all predictors, showing a strong positive correlation with attitudes (*r* = .74, *p* < .001) and a moderate correlation with subjective norms (*r* = .40, *p* < .001), perceived behavioural control (*r* = −.28, *p* < .001), gender (*r* = .30, *p* < .001), and religious influence (*r* = .40, *p* < .001). Attitudes and subjective norms were moderately correlated (*r* = .41, *p* < .001), while perceived behavioural control showed small negative correlations with both attitudes (*r* = −.23, *p* < .001) and subjective norms (*r* = −.13, *p* = .029).

A multiple linear regression with willingness to participate in abortion care as criterion, and abortion attitudes, subjective norms and perceived behavioural control as predictors explained 57.4% of the variance in willingness to participate in abortion care, *F*(5, 222) = 62.06, *p* < .001, adjusted *R*² = .574. Attitudes towards abortion (*B* = 0.50, BCa 95% CI [0.39, 0.59], *p* < .001, β = .68) and subjective norms (*B* = 0.10, BCa 95% CI [0.01, 0.20], *p* = .038, β = .11) were positively associated with willingness to participate in abortion care in the future. Perceived behavioural control was negatively associated with willingness to participate in abortion care (*B* = −0.05, BCa 95% CI [−0.08, −0.01], *p* = .008, β = −.11).

## Discussion

This study assessed the attitudes towards abortion in a sample of German medical students, and identified factors associated with attitudes towards abortion and willingness to participate in abortion care based on intergroup contact theory and the theory of planned behaviour. The results reveal generally positive attitudes among medical students. Although students expressed high willingness to engage in abortion care in the future, this willingness diminished with increased involvement in care, corroborating findings by Cohen et al. [42].

Gender and religiosity were significantly associated with abortion attitudes. Female students held more positive views on abortion than male students, while higher religiosity was associated with more negative attitudes, consistent with findings by Osborne et al. [25]. The study also explored intergroup effects, finding that participants with direct contact with abortion-seeking individuals showed a non-significant trend towards more positive attitudes. The expected indirect mediation effect between attitudes and contact through knowledge, perspective-taking and fear of stigmatisation was also not confirmed in our sample. Fear of stigmatisation and perceived knowledge were strongly associated with attitudes towards abortion, with fear of stigmatisation showing the most substantial association.

The negative associations between perceived knowledge and attitudes, and between perceived behavioural control and willingness to provide abortions, were unexpected and not fully consistent with the assumptions of intergroup contact theory and the theory of planned behaviour. Several explanations are possible. The measure of perceived knowledge reflects participants’ confidence in their knowledge and their perceived educational exposure rather than objective knowledge, making it susceptible to individual differences in self-assessment. For example, students with greater awareness of the legal, ethical, and clinical complexities surrounding abortion may rate their own knowledge more critically than students with more limited insight, resulting in lower perceived knowledge despite greater actual understanding. Conversely, less informed participants may overestimate their knowledge [43]. The construct of perceived behavioural control in our study may have captured perceived external barriers to providing abortion care, such as legal restrictions, limited training opportunities, institutional barriers, or anticipated stigma, rather than confidence in one's own abilities alone. Consequently, students who had reflected more extensively on the practical realities of abortion provision may have reported lower perceived behavioural control despite expressing generally favourable attitudes toward abortion.

Our findings regarding fear of stigmatisation as a strong predictor of attitudes and a barrier to provide abortion care are consistent with national and international research [10,13,16,44,45]. Interventions aiming at altering attitudes and increasing abortion care participation should focus on the reduction of abortion stigma, including the stigmatisation of abortion care providers. Evidence from previous studies indicates that educational interventions, such as guided workshops and open dialogue about abortion experiences can reduce stigma among healthcare providers [15,46].

This study also supports and quantitatively complements recent qualitative findings regarding the relevance of knowledge for medical students’ willingness to participate in abortion care [10,13]. The low perceived knowledge about abortion identified in this study – especially among those with more positive attitudes – highlights the need to address knowledge gaps among medical students. Evidence suggests that exposure to abortion procedures and interaction with individuals seeking abortions can positively influence attitudes [10,20]. Thus, integrating comprehensive abortion education into medical curricula may help bridge these gaps and better prepare students to deliver high-quality, evidence-based abortion care.

Equipping medical students with comprehensive clinical training in abortion care not only enhances their ability to provide safe, evidence-based care, but could also empower them to serve as influential advocates for progressive reproductive health policies. When physicians communicate with patients about abortion as a compassionate and patient-centred aspect of care, public attitudes tend to improve, and support for abortion restrictions declines [47]. In this way, clinical engagement and communication can contribute to a broader social environment that is more open to future legal and policy reforms.

The results indicate that participating medical students are open to engage in abortion care in Germany. Consistent with international research, this study aligns with previous findings showing a strong positive association between medical students’ attitudes towards abortion and their willingness to perform or support abortion care in the future [22,23]. However, the results contrast with Le et al.’s findings who reported that perceived behavioural control was the strongest predictor of behavioural intention to provide abortion care, with attitudes being the weakest predictor [26]. One possible explanation might be the difference in the study populations. Le et al. surveyed practising physicians, most of whom were between the age of 40 and 59 and had substantial professional experience. In contrast, our sample consisted of students with little or no clinical experience. Without relevant professional experience, students may rely more on their personal attitudes when forming behavioural intentions, whereas for practising physicians, intentions may be influenced more strongly by perceived behavioural control, reflecting their confidence in performing the behaviour, and by subjective norms related to their professional environment. The strong association between abortion attitudes and willingness to provide abortion care may also, in part, reflect conceptual overlap between the measures. While the AAS assesses general attitudes about abortion, willingness to provide abortion care reflects the behavioural expression of these attitudes. Although willingness constitutes a distinct construct by assessing professional behavioural intentions, it is theoretically expected to be strongly influenced by underlying attitudes toward abortion [21]. However, it is crucial to acknowledge that behavioural intentions do not always translate into actual practices [21]. The decrease of clinics and practices providing abortion care in Germany suggests that systemic and political barriers may hinder the realisation of these intentions. Although comprehensive abortion care is included in the list of essential health care services and recognised by World Health Organization as a fundamental human right [9], it remains inadequately implemented in Germany – particularly in comparison to other Western industrialised nations. Comparative international examples demonstrate potential pathways for reform: in Canada, complete decriminalisation of abortion has contributed to its normalisation and to improved access to care [48]. Fiala et al. describe how the absence of conscientious objection laws in three Nordic countries has helped to reduce the stigma associated with the procedure [49]. These findings suggest that changes in law and policy to better integrate abortion care into healthcare services could also potentially foster a more accepting societal context regarding abortion care in Germany. It remains to be seen whether political actors will be willing to support the necessary reforms in the coming years, or whether the medical profession will need to take the lead in actively advocating for equitable and nationwide access to abortion care.

### Strengths and limitations

The study has several key strengths. Its theoretical foundation, based on intergroup contact theory and the theory of planned behaviour, is robust and well-supported by prior research [17,18,21]. The careful translation and adaptation of scales and items to fit the study population's cultural and linguistic context minimise potential biases. Moreover, the nationwide survey reached participants from a broad spectrum of German medical schools. Our sample size was sufficiently large to allow for the detection of potential effects.

However, some limitations must be considered. As the first quantitative study of its kind in Germany, its exploratory character must be taken into consideration. The survey utilised a combination of established scales and self-developed items due to the lack of validated instruments, resulting in variable internal consistency. Thus, scales assessing constructs such as contact, perceived knowledge, and perceived behavioural control require further validation in future studies. Results that relate to these constructs should therefore be interpreted with appropriate caution. For instance, the scale measuring perceived knowledge assessed participants’ subjective perceptions of their knowledge as well as educational preparedness rather than objective factual knowledge. Specifically, it included items on perceived educational exposure (e.g., whether abortion-related topics were covered in the medical curriculum) as well as self-assessments of knowledge (e.g., whether participants felt they knew what they needed to know about abortion). The cross-sectional design limits the ability to infer causality from the observed associations. Additionally, our recruitment strategy through student councils, the voluntary survey participation and the absence of monetary incentives may have led to selective participation, potentially skewing results towards participants with positive attitudes and specific interest in gynaecology. Given that the number of female participants (82.8%) exceeded their proportion within the broader medical student population (64.6%) [34], and that certain federal states were underrepresented, the representativeness of the sample was not achieved. The study sample was predominantly female with positive attitudes towards abortion, suggesting a particularly engaged and reflective group of participants. This may also help explain why our hypotheses based on the contact hypothesis and the associated mediation effect were not supported. Although participants generally held positive attitudes towards abortion, they reported limited contact with individuals who have had an abortion. The restricted variability in these constructs may also have reduced our ability to detect the hypothesised effects. Consequently, our findings cannot be generalised to the general population of medical students. The developed survey requires validation in a more representative sample that reflects the composition of German medical students to obtain a more accurate picture of their attitudes and to derive more precise measures.

## Conclusions

This study represents the first quantitative assessment of attitudes towards abortion in a sample of medical students in Germany, revealing overall positive attitudes. It indicates a possible association of these attitudes and factors like fear of stigmatisation and perceived knowledge with the willingness to participate in abortion care. The study emphasises the importance of reducing the stigmatisation of individuals seeking abortions as well as abortion care providers and enhancing medical education on abortion within the medical curriculum.

Future research should focus on developing low-threshold interventions to overcome existing barriers for medical students to participate in abortion care, thereby improving access to high-quality abortion care in Germany.

## Supporting information

S1 Appendix Reporting checklist for cross sectional study. (DOCX)

S2 Appendix Items of the online survey and descriptive data of all items. (DOCX)

S3 Appendix Federal states of contacted medical schools and correlations of variables. (DOCX)
