# Supplementary material for: Who will step up? A cross-sectional study on factors associated with medical students’ willingness to provide abortion care
Source: PLoS One. 2026 Nov 6;21(11):e0362064. doi: 10.1371/journal.pone.0362064 (PMC13742952; doi:10.1371/journal.pone.0362064)
Supplement: Supplementary file 2 [file pone.0362064.s002.docx]

**S2 Appendix.** **Items of the online survey and descriptive data of all items.**

Below, all scales and items used in the online survey are listed and descriptive data (absolute and relative frequencies) are presented. As mentioned in the main manuscript, some of the scales below are already validated in English or German, some scales were not validated or single items were used. Furthermore, we developed single items. All items, which were not self-developed or already available in English were translated into German by the study team via a team translation protocol. However, as only some of the scales are validated, the English as well as the German versions have to be used with caution and original references of the items and scales have to be checked.

The analyses presented in the main manuscript included only selected constructs and scales. Items or scales that have not been analysed and thus have not been reported in the main manuscript are marked with an asterisk (*).

| **Attitudes towards abortion: Abortion Attitude Scale**  *This is a validated scale, which was translated and adapted by the study team. The English version is a translation of the items used in the German survey and not validated.*  *Reference: Sloan, L. A. (1983). Abortion Attitude Scale. Health Education, 14(3), 41–42. https://doi.org/10.1080/00970050.1983.10614403* | | | | | | |
| --- | --- | --- | --- | --- | --- | --- |
| *Introduction:* The following questions concern your attitudes toward abortion.  This is not a test; there are no right or wrong answers. Please answer as honestly as possible. The statements focus on your views regarding abortions within the first three months of pregnancy performed by a qualified medical professional. Please indicate how strongly you agree or disagree with each statement by selecting one response option.  Please answer each statement. Your responses are anonymous and cannot be linked back to you. Only study personnel will have access to your responses.  *(Im Folgenden geht es um Ihre Einstellungen zum Thema Schwangerschaftsabbruch:*  *Dies ist kein Test. Es gibt keine richtigen oder falschen Antworten. Bitte antworten Sie so ehrlich wie möglich. In den Aussagen geht es um Ihre Ansichten über Schwangerschaftsabbrüche während der ersten drei Monate der Schwangerschaft durch eine qualifizierte medizinische Person. Bitte geben Sie an, wie sehr Sie einer Aussage zustimmen oder sie ablehnen, indem Sie eine Antwortoption ankreuzen. Niemand wird Ihre Antwort ohne Erlaubnis sehen können.)* | | | | | | |
|  | Strongly disagree  *(Stimme überhaupt nicht zu)* | Disagree  *(Stimme nicht zu)* | Slightly disagree  *(Stimme eher nicht zu)* | Slightly agree  *(Stimme eher zu)* | Agree  *(Stimme zu)* | Strongly agree  *(Stimme vollkomme zu)* |
|  | n (%) | | | | | |
| In Germany, abortions should be banned under all circumstances.  *(In Deutschland sollten Schwangerschaftsabbrüche unter allen Umständen verboten werden.)* | 205 (88) | 15 (6.4) | 5 (2.1) | 3 (1.3) | 4 (1.7) | 1 (0.4) |
| Abortion is a good option to end an unwanted pregnancy.  *(Ein Schwangerschaftsabbruch ist eine gute Möglichkeit, eine ungewollte Schwangerschaft zu beenden.)* | 7 (3.0) | 5 (2.1) | 13 (5.6) | 28 (12) | 53 (22.7) | 127 (54.5) |
| A person should feel obligated to give birth to the child that was conceived.  *(Eine Person sollte sich verpflichtet fühlen, das Kind zu gebären, das gezeugt wurde.)* | 148 (63.5) | 38 (16.3) | 16 (6.9) | 18 (7.7) | 9 (3.9) | 4 (1.7) |
| Abortions are wrong under all circumstances.  *(Schwangerschaftsabbrüche sind unter allen Umständen falsch.)* | 193 (82.8) | 25 (10.7) | 8 (3.4) | 3 (1.3) | 3 (1.3) | 1 (0.4) |
| A fetus is only a person when it can live outside the body of the pregnant person.  *(Ein Fötus ist erst eine Person, wenn er außerhalb des Körpers der schwangeren Person leben kann.)* | 24 (10.3) | 44 (18.9) | 57 (24.5) | 42 (18.0) | 46 (19.7) | 20 (8.6) |
| The decision to have an abortion should lie with the pregnant person.  *(Die Entscheidung für einen Schwangerschaftsabbruch sollte bei der schwangeren Person liegen.)* | 1 (0.4) | 2 (0.9) | 6 (2.6) | 23 (9.9) | 51 (21.9) | 150 (64.4) |
| Every conceived child has the right to be born.  *(Jedes gezeugte Kind hat das Recht, geboren zu werden.)* | 60 (25.9) | 67 (28.9) | 54 (23.3) | 31 (13.4) | 8 (3.4) | 12 (5.2) |
| A pregnant person who does not want a child should be encouraged to have an abortion.  *(Eine schwangere Person, die kein Kind haben möchte, sollte zu einem Schwangerschaftsabbruch ermutigt werden.)* | 35 (15.1) | 34 (14.7) | 89 (38.4) | 50 (21.6) | 17 (7.3) | 7 (3.0) |
| Abortion should be considered as killing a human being.  *(Ein Schwangerschaftsabbruch sollte als Tötung eines Menschen gelten.)* | 170 (73) | 28 (12) | 14 (6) | 13 (5.6) | 4 (1.7) | 4 (1.7) |
| People should not look down on those who choose to have an abortion.  *(Menschen sollten nicht auf diejenigen herabschauen, die sich für Schwangerschaftsabbrüche entscheiden.)* | 8 (3.4) | 0 (0.0) | 3 (1.3) | 7 (3.0) | 30 (12.9) | 185 (79.4) |
| Abortion should be an easily accessible option for pregnant minors.  *(Ein Schwangerschaftsabbruch sollte eine leicht zugängliche Alternative für schwangere Minderjährige sein.)* | 8 (3.4) | 19 (8.2) | 35 (15) | 50 (21.5) | 46 (19.7) | 75 (32.2) |
| No one should have power over the life or death of a foetus.  *(Niemand sollte die Macht über das Leben oder den Tod eines Fötus haben.)* | 98 (42.6) | 69 (30.0) | 46 (20.0) | 11 (4.8) | 3 (1.3) | 3 (1.3) |
| Unwanted children should not be brought into the world.  *(Ungewollte Kinder sollten nicht auf die Welt gebracht werden.)* | 29 (12.5) | 45 (19.4) | 50 (21.6) | 65 (28.0) | 27 (11.6) | 16 (6.9) |
| A foetus should be considered a person from the moment of conception.  *(Ein Fötus sollte ab dem Zeitpunkt der Empfängnis als Person betrachtet werden.)* | 99 (42.5) | 56 (24) | 47 (20.2) | 20 (8.6) | 4 (1.7) | 7 (3.0) |

| **Additional scale to assess attitudes towards abortion I***  *This is a non-validated scale, which was translated and adapted by the study team. The English version is a translation of the items used in the German survey and not validated.*  *Reference: Myran, D. T., Carew, C. L., Tang, J., Whyte, H. & Fisher, W. A. (2015). Medical students' intentions to seek abortion training and to provide abortion services in future practice. Journal of Obstetrics and Gynaecology Canada, 37(3), 236–244.*  *Pace, L., Sandahl, Y., Backus, L., Silveira, M. & Steinauer, J. (2008). Medical Students for Choice's Reproductive Health Externships: impact on medical students' knowledge, attitudes and intention to provide abortions. Contraception, 78(1), 31–35. https://doi.org/10.1016/j.contraception.2008.02.008* | | | | | |
| --- | --- | --- | --- | --- | --- |
| *Introduction:* Do you personally think it is wrong or not wrong for a woman to have an abortion in the following circumstances?*  *(Halten Sie es persönlich für richtig oder falsch, eine Schwangerschaft unter folgenden Umständen abzubrechen…)* | | | | | |
|  | Oppose a lot  *(Lehne stark ab)* | Oppose somewhat  *(Lehne teilweise ab)* | Neither support nor oppose  *(Weder Ablehnung noch Befürwortung)* | Oppose somewhat  *(Befürworte teilweise)* | Oppose a lot  *(Befürworte stark)* |
|  | n (%) | | | | |
| Under all circumstances up to the 9th week of pregnancy*  *(Unter allen Umständen bis zur 9. Woche der Schwangerschaft)* | 10 (4.3) | 8 (3.4) | 18 (7.8) | 45 (19.4) | 151 (65.1) |
| Under all circumstances up to the 14th week of pregnancy*  *(Unter allen Umständen bis zur 14. Woche der Schwangerschaft)* | 11 (4.7) | 26 (11.2) | 24 (10.3) | 79 (33.9) | 93 (39.9) |
| Under all circumstances up to the 24th week of pregnancy*  *(Unter allen Umständen bis zur 24. Woche der Schwangerschaft)* | 37 (15.9) | 72 (31.0) | 41 (17.7) | 59 (25.4) | 23 (9.9) |
| In emergencies in which the pregnancy endangers the life of the pregnant person*  *(In Notfällen, in denen die Schwangerschaft das Leben der schwangeren Person gefährdet)* | 0 (0.0) | 0 (0.0) | 2 (0.9) | 10 (4.3) | 221 (94.8) |
| If the pregnancy is the result of rape*  *(Wenn die Schwangerschaft die Folge einer Vergewaltigung ist)* | 2 (0.9) | 2 (0.9) | 10 (4.3) | 29 (12.4) | 190 (81.5) |
| If the pregnancy is the result of incest*  *(Wenn die Schwangerschaft die Folge von Inzest ist)* | 7 (3.0) | 10 (4.3) | 29 (12.5) | 50 (21.6) | 136 (58.6) |
| If the foetus has hereditary, non-lethal impairments (e.g. Down syndrome)*  *(Wenn der Fötus erblich bedingte, nicht tödliche Beeinträchtigungen hat (z.B. Down Syndrom))* | 22 (9.4) | 57 (24.5) | 64 (27.5) | 62 (26.6) | 28 (12) |
| Under no circumstances*  *(Unter keinen Umständen)* | 190 (81.9) | 19 (8.2) | 14 (6.0) | 4 (1.7) | 5 (2.2) |
| Under any circumstances*  *(Unter allen Umständen)* | 45 (19.4) | 44 (19.0) | 50 (21.6) | 64 (27.6) | 29 (12.5) |
| For financial reasons*  *(Aus finanziellen Gründen)* | 29 (12.6) | 39 (17.0) | 50 (21.7) | 81 (35.2) | 31 (13.5) |
| When a child is not compatible with a career*  *(Wenn ein Kind nicht mit der Karriere vereinbar ist)* | 36 (15.5) | 39 (16.7) | 50 (21.5) | 74 (31.8) | 34 (14.6) |
| If a child is not compatible with school/training/studies*  *(Wenn ein Kind nicht mit der Schule/der Ausbildung/dem Studium vereinbar ist)* | 24 (10.3) | 31 (13.4) | 50 (21.6) | 82 (35.3) | 45 (19.4) |
| If the sex of the fetus does not correspond to the parents' wishes*  *(Wenn das Geschlecht des Fötus nicht dem Wunsch der Eltern entspricht)* | 204 (87.6) | 20 (8.6) | 8 (3.4) | 1 (0.4) | 0 (0.0) |
| If the parents do not want the child*  *(Wenn die Eltern das Kind nicht haben möchten)* | 22 (9.4) | 30 (12.9) | 34 (14.6) | 71 (30.5) | 76 (32.6) |

| **Additional scale to assess attitudes towards abortion II***  *This is a non-validated scale, which was translated and adapted by the study team. The English version is a translation of the items used in the German survey and not validated.*  *Reference: Schmuhl, N. B., Rice, L. W., Wautlet, C. K. & Higgins, J. A. (2021). Physician attitudes about abortion and their willingness to consult in abortion care at a Midwestern academic medical center. Contraception, 104(3), 278–283. https://doi.org/10.1016/j.contraception.2021.04.030* | | | | | |
| --- | --- | --- | --- | --- | --- |
| *Introduction:* How much do you support or oppose:*  *(Wie stark stimmen Sie zu oder lehnen Sie ab:)* | | | | | |
|  | Oppose a lot  *(Lehne stark ab)* | Oppose somewhat  *(Lehne teilweise ab)* | Neither support nor oppose  *(Weder Ablehnung noch Befürwortung)* | Support somewhat  *(Befürworte teilweise)* | Support a lot  *(Befürworte stark)* |
|  | n (%) | | | | |
| Medication abortion*  *(Medikamentöse Schwangerschaftsabbrüche)* | 3 (1.3) | 5 (2.2) | 27 (11.6) | 53 (22.8) | 144 (62.1) |
| Surgical abortion*  *(Operative Schwangerschaftsabbrüche)* | 3 (1.3) | 4 (1.7) | 32 (13.8) | 69 (29.7) | 124 (53.4) |

| **Additional scale to assess attitudes towards abortion III***  *These are self-developed items. The English version is a translation of the items used in the German survey and not validated.* | | | | | |
| --- | --- | --- | --- | --- | --- |
| *Introduction:* How much do you support or oppose:*  *(Wie stark stimmen Sie zu oder lehnen Sie ab:)* | | | | | |
|  | Oppose a lot  *(Lehne stark ab)* | Oppose somewhat  *(Lehne teilweise ab)* | Neither support nor oppose  *(Weder Ablehnung noch Befürwortung)* | Support somewhat  *(Befürworte teilweise)* | Support a lot  *(Befürworte stark)* |
|  | n (%) | | | | |
| A mandatory counselling session at a counselling center.*  *(Ein verpflichtendes Beratungsgespräch in einer Beratungsstelle.)* | 8 (3.4) | 31 (13.3) | 12 (5.2) | 53 (22.7) | 129 (55.4) |
| A mandatory waiting period between a consultation and an abortion.*  *(Eine verpflichtende Wartezeit zwischen einem Beratungsgespräch und dem Schwangerschaftsabbruch.)* | 22 (9.5) | 42 (18.1) | 19 (8.2) | 74 (31.9) | 75 (32.3) |
| The doctor's personal values regarding abortion should be incorporated into the consultation and treatment.*  *(Die persönliche Wertvorstellung des:der Ärzt:in zum Schwangerschaftsabbruch sollte in die Beratung und Behandlung einfließen.)* | 192 (82.4) | 26 (11.2) | 5 (2.1) | 9 (3.9) | 1 (0.4) |
| Doctors should have the right to refuse to perform an abortion.*  *(Ärzt:innen sollten das Recht haben, die Durchführung eines Schwangerschaftsabbruches zu verweigern.)* | 44 (18.9) | 41 (17.6) | 15 (6.4) | 47 (20.2) | 86 (36.9) |

| **Perceived behavioural control**  *These are self-developed items. The English version is a translation of the items used in the German survey and not validated.* | | | | | |
| --- | --- | --- | --- | --- | --- |
| *Introduction:* Please indicate whether you agree or disagree with the following statement.  *(Bitte geben Sie an, ob Sie der folgenden Aussage zustimmen oder nicht zustimmen.)* | | | | | |
|  | Disagree  *(Stimme nicht zu)* | Partly disagree  *(Stimme teilweise nicht zu)* | Neither agree nor disagree  *(Weder noch)* | Partly agree  *(Stimme teilweise zu)* | Strongly agree  *(Stimme stark zu)* |
|  | n (%) | | | | |
| Whether I perform abortions in my future profession will depend solely on my own decision.  *(Ob ich in meinem späteren Berufsfeld Schwangerschaftsabbrüche durchführe, wird allein von meiner eigenen Entscheidung abhängen.)* | 34 (14.7) | 33 (14.3) | 27 (11.7) | 67 (29.0) | 70 (30.3) |
| The responsibility for providing adequate care for those who wish to have an abortion lies with politicians.  *(Die Verantwortung für eine ausreichende Versorgung bei Wunsch nach einem Schwangerschaftsabbruch liegt bei der Politik.)* | 9 (3.9) | 25 (10.7) | 24 (10.3) | 103 (44.2) | 72 (30.9) |
| The medical profession is responsible for providing adequate care in the event of a request for an abortion.  *(Die Verantwortung für eine ausreichende Versorgung bei Wunsch nach einem Schwangerschaftsabbruch liegt bei der Ärzteschaft.)* | 12 (5.2) | 22 (9.4) | 29 (12.4) | 140 (60.1) | 30 (12.9) |

| **Subjective norms**  *This is a non-validated scale, which was translated and adapted by the study team. The English version is a translation of the items used in the German survey and not validated.*  *Reference: Schmuhl, N. B., Rice, L. W., Wautlet, C. K. & Higgins, J. A. (2021). Physician attitudes about abortion and their willingness to consult in abortion care at a Midwestern academic medical center. Contraception, 104(3), 278–283. https://doi.org/10.1016/j.contraception.2021.04.030* | | | | | |
| --- | --- | --- | --- | --- | --- |
| *Introduction:* Please tell us if the following groups of people oppose or support unrestricted access to abortion?  *(Bitte geben Sie an, ob die folgenden Personen einen uneingeschränkten Zugang zu Schwangerschaftsabbrüchen ablehnen oder unterstützen.)* | | | | | |
|  | Oppose a lot  *(Lehnen stark ab)* | Oppose somewhat  *(Lehnen teilweise ab)* | Neither oppose nor support  *(Weder Ablehnung noch Befürwortung)* | Support somewhat  *(Befürworten teilweise)* | Support a lot  *(Befürworten stark)* |
|  | n (%) | | | | |
| Yourself*  *(Sie selbst)* | 9 (3.9) | 15 (6.4) | 6 (2.6) | 53 (22.7) | 150 (64.4) |
| Family members  *(Familienmitglieder)* | 10 (4.3) | 39 (16.9) | 21 (9.1) | 103 (44.6) | 58 (25.1) |
| Friends  *(Freund:innen)* | 5 (2.2) | 8 (3.4) | 11 (4.7) | 87 (37.5) | 121 (52.2) |
| Your classmates in your degree program  *(Ihre Kommiliton:innen in Ihrem Studiengang)* | 1 (0.4) | 9 (3.9) | 32 (13.9) | 122 (52.8) | 67 (29.0) |
| Supervisors at your university clinic (e.g., chief physicians, clinic leadership)  *(Führungspersonen in Ihrer Universitätsklinik (zum Beispiel Chefärzt:innen, Klinikleitung))* | 2 (0.9) | 34 (15.2) | 104 (46.4) | 69 (30.8) | 15 (6.7) |
| Medical personnel at your university clinic (e.g., assistant doctors)  *(ärztliches Personal in Ihrer Universiätsklinik (zum Beispiel Assistenzärzt:innen))* | 0 (0.0) | 26 (11.7) | 83 (37.2) | 90 (40.4) | 24 (10.8) |

| **Willingness to participate in abortion care**  *This is a non-validated scale, which was translated and adapted by the study team. The English version is a translation of the items used in the German survey and not validated.*  *Reference: Cohen, P., Mayhew, J., Gishen, F., Potts, H. W. W., Lohr, P. A. & Kavanagh, J. (2021). What should medical students be taught about abortion? An evaluation of student attitudes towards their abortion teaching and their future involvement in abortion care. BMC Medical Education, 21(1), 4. https://doi.org/10.1186/s12909-020-02414-9* | | | | |
| --- | --- | --- | --- | --- |
| *Introduction:* In your future work as a physician, would you be willing to participate in the following aspects of abortion care (assuming you had the necessary training and experience)?  *Wären Sie in Ihrer zukünftigen Tätigkeit als Ärzt:in bereit, an den folgenden Aspekten in der Versorgung von Schwangerschaftsabbrüchen beteiligt zu sein (angenommen, Sie hätte die nötige Ausbildung und Erfahrung)?* | | | | |
|  | No, under no circumstances  *(Nein, unter keinen Umständen)* | Undecided  *(Unentschlossen)* | Yes, but only under certain circumstances  *(Ja, aber nur unter bestimmten Umständen)* | Yes, under all legal circumstances  *(Ja, unter allen legalen Umständen)* |
|  | n (%) | | | |
| Counselling on abortion options  *Beratung über Möglichkeiten des Umgangs mit der Schwangerschaft* | 2 (0.9) | 2 (0.9) | 11 (4.7) | 218 (93.6) |
| Referral to a colleague who can perform the abortion  *(Überweisung zu eine:r Kolleg:in, der:die den Schwangerschaftsabbruch durchführen kann)* | 7 (3) | 1 (0.4) | 21 (9) | 204 (87.6) |
| Performing medical (pharmaceutical) abortions  *(Durchführung medikamentöser Schwangerschaftsabbrüche)* | 13 (5.6) | 20 (8.6) | 36 (15.5) | 164 (70.4) |
| Performing surgical abortions  *(Durchführung operativer Schwangerschaftsabbrüche)* | 18 (7.7) | 31 (13.3) | 43 (18.5) | 141 (60.5) |

| **Contact with individuals seeking abortions**  *These are self-developed items. The English version is a translation of the items used in the German survey and not validated.* | | | | | |
| --- | --- | --- | --- | --- | --- |
| *Introduction:* The following questions pertain to your experiences with abortion and contact with individuals who have had an abortion.  *(Im Folgenden geht es um Ihre Erfahrungen mit Schwangerschaftsbrüchen und Kontakt zu Personen, die einen Schwangerschaftsabbruch hatten.)* | | | | | |
|  | Not at all  *(Gar nicht)* | Rarely  *(Selten=* | Occasionally  *(Gelegentlich)* | Often  *(Häufig)* | Daily  *(Täglich)* |
|  | n (%) | | | | |
| How often do you have contact with people in your close circle of friends or family who have had an abortion?  *(Wie häufig haben Sie Kontakt zu Personen in Ihrem engeren Freundeskreis oder Ihrer Familie, die einen Schwangerschaftsabbruch hatten?)* | 146 (62.7) | 43 (18.5) | 25 (10.7) | 15 (6.4) | 4 (1.7) |
| How often do you have contact with people in your (extended) acquaintance circle who have had an abortion?  *(Wie häufig haben Sie Kontakt zu Personen aus Ihrem (erweiterten) Bekanntenkreis, die einen Schwangerschaftsabbruch hatten?)* | 105 (45.1) | 87 (37.3) | 33 (14.2) | 8 (3.4) | 0 (0.0) |
| How often have you encountered people during your studies (e.g., during an internship or practical year) who have had or wanted to have an abortion?  *(Wie häufig hatten Sie während Ihres Studiums (z.B. während eines Praktikums oder PJ) Kontakt zu Personen, die einen Schwangerschaftsabbruch vorgenommen haben oder vornehmen wollten lassen?)* | 107 (45.9) | 68 (29.2) | 43 (18.5) | 14 (6) | 1 (0.4) |

| **Perceived knowledge regarding abortion care**  *This is a non-validated scale, which was translated and adapted by the study team. The English version is a translation of the items used in the German survey and not validated.*  *Reference: Cohen, P., Mayhew, J., Gishen, F., Potts, H. W. W., Lohr, P. A. & Kavanagh, J. (2021). What should medical students be taught about abortion? An evaluation of student attitudes towards their abortion teaching and their future involvement in abortion care. BMC Medical Education, 21(1), 4. https://doi.org/10.1186/s12909-020-02414-9; Pace, L., Sandahl, Y., Backus, L., Silveira, M. & Steinauer, J. (2008). Medical Students for Choice's Reproductive Health Externships: impact on medical students' knowledge, attitudes and intention to provide abortions. Contraception, 78(1), 31–35. https://doi.org/10.1016/j.contraception.2008.02.008* | | | | | |
| --- | --- | --- | --- | --- | --- |
| *Introduction:* We would like to know whether and how the topic of abortion is integrated into your medical education.  *(Im Folgenden möchten wir gerne wissen, ob und wie das Thema Schwangerschaftsabbruch in Ihrer medizinischen Ausbildung integriert ist).* | | | | | |
|  | Definitely no  *(Auf keinen Fall)* | Probably no  *(Eher nein)* | Undecided  *(Unentschlossen)* | Probably yes  *(Eher ja)* | Definitely yes  *(Auf jeden Fall)* |
|  | n (%) | | | | |
| I think it is important that medical students learn something about the performance and care of abortions.*  *(Ich finde es wichtig, dass Medizinstudierende etwas über die Durchführung und Versorgung von Schwangerschaftsabbrüchen lernen.)* | 0 (0.0) | 6 (2.6) | 5 (2.1) | 29 (12.4) | 193 (82.8) |
| In my studies so far, abortion has been discussed apart from medical ethics (or ethical and legal aspects).  *(Schwangerschaftsabbrüche wurden in meinem bisherigen Studium, abseits der Medizinethik (oder: ethischer und rechtlicher Aspekte), thematisiert.)* | 53 (23.0) | 92 (40.0) | 27 (11.7) | 43 (18.7) | 15 (6.5) |
| My medical education at the university has covered everything I need to know about abortion.  *(Meine medizinische Ausbildung an der Universität zum Thema Schwangerschaftsabbruch hat alles beinhaltet, was ich dazu wissen muss.)* | 82 (36.0) | 75 (32.9) | 57 (25.0) | 12 (5.3) | 2 (0.9) |
| During my medical studies, I received training or instruction on abortion outside the regular curriculum (e.g., in a papaya workshop by Medical Students for Choice).  *(Ich hatte während meines Medizinstudiums außerhalb des normalen Lehrplans Schulungen oder Unterweisungen zu Schwangerschaftsabbrüchen (zum Beispiel in einem Papaya-Workshop der Medical Students for Choice).)* | 96 (42.3) | 61 (26.9) | 33 (14.5) | 20 (8.8) | 17 (7.5) |
| I feel sufficiently informed about abortion methods and could counsel patients on the topic.  *(Ich fühle mich ausreichend über die Methoden des Schwangerschaftsabbruchs informiert, und könnte Patient:innen dazu beraten.)* | 79 (34.1) | 88 (37.9) | 38 (16.4) | 24 (10.3) | 3 (1.3) |

| **Fear of stigmatisation: Abortion Providers Stigma Scale**  *This is a validated scale, which was translated and adapted by the study team. The English version is a translation of the items used in the German survey and not validated.*  *Reference: Martin, L. A., Hassinger, J. A., Seewald, M. & Harris, L. H. (2018). Evaluation of abortion stigma in the workforce: Development of the Revised Abortion Providers Stigma Scale. Womens Health Issues, 28(1), 59–67. https://doi.org/10.1016/j.whi.2017.10.004* | | | | | |
| --- | --- | --- | --- | --- | --- |
| *Introduction:* The following questions concern perceived stigma related to providing abortion services.  Imagine the experiences you might have if you were involved in providing abortion care. How often do you think you would find yourself in the following situations? Please relate these statements to aspects of your work related to abortion services.  *(Im Folgenden geht es um die wahrgenommene Stigmatisierung im Zusammenhang mit der Durchführung von Schwangerschaftsbrüchen.*  *Bitte stellen Sie sich vor, welche Erfahrungen Sie machen würden, wenn Sie in der Versorgung von Schwangerschaftsabbrüchen tätig wären. Was glauben Sie, wie häufig würden Sie sich in den folgenden Aussagen wiederfinden?*  *Bitte beziehen Sie sich bei der Beantwortung auf Aspekte Ihrer Arbeit im Zusammenhang mit der Durchführung von Schwangerschaftsabbrüchen.)* | | | | | |
|  | Never  *(Nie)* | Rarely  *(Selten)* | Sometimes  *(Manchmal)* | Often  *(Oft)* | All of the time  *(Immer)* |
|  | n (%) | | | | |
| People's reactions to my being an abortion worker would make me keep to myself.  *(Die Reaktion anderen Personen darauf, dass ich Schwangerschaftsabbrüche durchführe, würden mich dazu bringen, mich bedeckt zu halten.)* | 42 (18) | 67 (28.8) | 80 (34.3) | 40 (17.2) | 4 (1.7) |
| I would feel like if I tell people about my work they would ONLY see me as an abortion worker.  *(Ich hätte das Gefühl, wenn ich anderen Personen von meiner Arbeit erzähle, werden sie mich nur als jemanden betrachten, der:die Schwangerschaftsabbrüche durchführt.)* | 39 (16.7) | 72 (30.9) | 85 (36.5) | 33 (14.2) | 4 (1.7) |
| I would worry about telling people I worked in abortion care.  *(Ich würde mir Sorgen machen, anderen Personen zu erzählen, dass ich Schwangerschaftsabbrüche durchführe.)* | 49 (21) | 90 (38.6) | 65 (27.9) | 20 (8.6) | 9 (3.9) |
| It would bother me if people in my neighborhood knew that I worked in abortion care.  *(Es würde mich stören, wenn Personen in meiner Nachbarschaft wüssten, dass ich Schwangerschaftsabbrüche durchführe.)* | 97 (41.6) | 74 (31.8) | 41 (17.6) | 19 (8.2) | 2 (0.9) |
| I would avoid telling people what I did for a living.  *(Ich würde es vermeiden, Personen zu erzählen, dass ich meinen Lebensunterhalt unter anderem mit der Durchführung von Schwangerschaftsabbrüchen verdiene.)* | 66 (28.3) | 69 (29.6) | 48 (20.6) | 45 (19.3) | 5 (2.1) |
| I would be afraid that if I told people I worked in abortion care I could put myself, or my loved ones, at risk for violence.  *(Ich hätte Angst, dass ich mich selbst oder meine Angehörigen Anfeindungen aussetze, wenn ich erzählte, dass ich Schwangerschaftsabbrüche durchführe.)* | 37 (15.9) | 63 (27.0) | 80 (34.3) | 45 (19.3) | 8 (3.4) |
| I would feel that disclosing my abortion work was not worth the potential hassle that could result.  *(Ich hätte das Gefühl, dass eine Offenlegung meiner Arbeit den möglichen Ärger nicht wert wäre, der sich daraus ergeben könnte.)* | 82 (35.2) | 66 (28.3) | 53 (22.7) | 27 (11.6) | 5 (2.1) |
| I would be afraid of how people will react if they found out about my work in abortion care.  *(Ich hätte Angst, wie Personen reagieren, wenn sie herausfinden, dass ich Schwangerschaftsabbrüche durchführe.)* | 58 (24.9) | 85 (36.5) | 61 (26.2) | 26 (11.2) | 3 (1.3) |
| I would feel the need to hide my work in abortion care from my friends.  *(Ich hätte das Gefühl, dass ich meinen Freund:innen verheimlichen muss, dass ich Schwangerschaftsabbrüche durchführe.)* | 175 (75.4) | 44 (19.0) | 3 (1.3) | 8 (3.4) | 2 (0.9) |
| I would find it hard to tell people I worked in abortion care.  *(Es würde mir schwerfallen, anderen davon zu erzählen, dass ich Schwangerschaftsabbrüche durchführe.)* | 75 (32.2) | 82 (35.2) | 52 (22.3) | 20 (8.6) | 4 (1.7) |

| **Perspective-taking: Jefferson Scale of Physician Empathy – Students Version**  *This is a validated German scale comprising 20 items in total. For this survey, 10 items from the perspective-taking dimension have been used from this scale. The English version is a translation of the items used in the German survey and not validated.*  *Reference: Preusche, I. & Wagner-Menghin, M. (2013). Rising to the challenge: Cross-cultural adaptation and psychometric evaluation of the adapted German version of the Jefferson Scale of Physician Empathy for Students (JSPE-S). Advances in Health Sciences Education, 18(4), 573–587. https://doi.org/10.1007/s10459-012-9393-9* | | | | | | |
| --- | --- | --- | --- | --- | --- | --- |
| *Introduction:* The following questions concern communication between patients and physicians. Please indicate the extent to which you agree or disagree with each of the following statements.  *(Im Folgenden geht es um die Kommunikation zwischen Patient:innen und Ärzt:innen. Bitte geben Sie an, inwieweit Sie jeder der folgenden Aussagen zustimmen oder ablehnen.)* | | | | | | |
|  | Strongly disagree  *(Stimme überhaupt nicht zu)* | Disagree  *(Stimme nicht zu)* | Partly disagree  *(Stimme eher nicht zu)* | Partly agree  *(Stimme eher zu)* | Agree  *(Stimme zu)* | Strongly agree  *(Stimme vollkommen zu)* |
|  | n (%) | | | | | |
| Patients feel better when their physicians understand their feelings.  *(Patient:innen fühlen sich besser, wenn ihr:e Ärzt:in ihre Gefühle versteht.)* | 0 (0.0) | 0 (0.0) | 0 (0.0) | 6 (2.6) | 50 (21.5) | 177 (76) |
| Understanding body language is as important as verbal communication in physician-patient relationships.  *(In der Beziehung zwischen der:dem Ärzt:in und den Patient:innen ist das Verstehen der Körpersprache genauso wichtig wie die verbale Kommunikation.)* | 0 (0.0) | 2 (0.9) | 0 (0.0) | 21 (9.1) | 60 (25.9) | 149 (64.2) |
| Attentiveness to patients’ personal experiences does not influence treatment outcomes.  *(Es hat keinen Einfluss auf die Behandlungsergebnisse, wenn man die persönlichen Erfahrungen der Patient:innen berücksichtigt.)* | 141 (60.5) | 60 (25.8) | 27 (11.6) | 3 (1.3) | 2 (0.9) | 0 (0.0) |
| Physicians should try to stand in their patients’ shoes when providing care to them.  *(Ärzt:innen sollten versuchen sich in die Lage ihrer Patient:innen zu versetzen, wenn sie diese behandeln.)* | 0 (0.0) | 0 (0.0) | 2 (0.9) | 40 (17.2) | 80 (34.3) | 111 (47.6) |
| Patients value a physician’s understanding of their feelings, which is therapeutic in its own right.  *(Patient:innen schätzen das Verständnis der:des Ärzt:in für ihre Gefühle; dies allein hat schon einen therapeutischen Effekt.)* | 0 (0.0) | 0 (0.0) | 4 (1.7) | 25 (10.7) | 81 (34.8) | 123 (52.8) |
| Asking patients about what is happening in their personal lives is not helpful in understanding their physical complaints.  *(Es ist nicht hilfreich Patient:innen danach zu fragen, was gerade in ihrem Leben passiert, um ihre körperlichen Beschwerden zu verstehen.)* | 165 (70.8) | 54 (23.2) | 11 (4.7) | 1 (0.4) | 1 (0.4) | 1 (0.4) |
| Physicians should try to understand what is going on in their patients’ minds by paying attention to their non-verbal cues and body language.  *(Ärzt:innen sollten versuchen zu verstehen, was in den Köpfen ihrer Patient:innen vorgeht, indem sie auf ihre nonverbalen Hinweise und Körpersprache achten.)* | 1 (0.4) | 0 (0.0) | 1 (0.4) | 21 (9) | 72 (30.9) | 138 (59.2) |
| Empathy is a therapeutic skill without which the physician’s success is limited.  *(Empathie ist eine therapeutische Fertigkeit, ohne die der Erfolg einer:eines Ärzt:in eingeschränkt ist.)* | 0 (0.0) | 0 (0.0) | 1 (0.4) | 28 (12.1) | 61 (26.3) | 142 (61.2) |
| Physicians’ understanding of the emotional status of their patients, as well as that of their families is one important component of the physician-patient relationship.  *(Eine wichtige Komponente in der Beziehung zwischen der:dem Ärzt:in und den Patient:innen ist es, dass der:die Ärzt:in für das emotionale Befinden der PatientInnen und das ihrer Angehörigen Verständnis hat.)* | 0 (0.0) | 0 (0.0) | 1 (0.4) | 24 (10.3) | 74 (31.8) | 134 (57.5) |
| I believe that empathy is an important therapeutic factor in medical treatment.  *(Ich glaube, dass Empathie ein wichtiger therapeutischer Faktor in der medizinischen Behandlung ist.)* | 0 (0.0) | 0 (0.0) | 0 (0.0) | 13 (5.6) | 56 (24) | 164 (70.4) |

| **Factual knowledge regarding the topic of abortion care***  *These are self-developed items. The English version is a translation of the items used in the German survey and not validated.* | | | | | | | | |
| --- | --- | --- | --- | --- | --- | --- | --- | --- |
| Introduction: The following is about your knowledge on the subject of abortion.*  *(Im Folgenden geht es um Ihr Wissen zum Thema Schwangerschaftsbruch.)* | | | | | | | | |
| 1. Which method(s) of abortion are recommended by the WHO?*  *(Welche Methode(n) des Schwangerschaftsabbruchs werden von der WHO empfohlen?)* | | | | | | No *(Nein)* | Yes *(Ja)* | |
|  |  |  |  |  |  | n (%) | | |
| medical abortion up to the 12th week of pregnancy* *(medikamentöser Abbruch bis zur 12. Woche der Schwangerschaft)* | | | | | | 49 (21) | 184 (79) | |
| surgical abortion by means of suction (vacuum aspiration) up to the 14th week of pregnancy* *(operativer Abbruch mittels Absaugung (Vakuumaspiration) bis zur 14. Woche der Schwangerschaft)* | | | | | | 69 (29.6) | 164 (70.4) | |
| surgical abortion by means of curettage (scraping) up to the 14th week of pregnancy* *(operativer Abbruch mittels Kürettage (Ausschabung) bis zur 14. Woche der Schwangerschaft)* | | | | | | 105 (45.1) | 128 (54.9) | |
| Surgical abortion by means of dilatation & evacuation (D&E) from the 14th week of pregnancy* *(operativer Abbruch mittels Dilatation & Evakuierung (D&E) ab der 14. Woche der Schwangerschaft)* | | | | | | 177 (76) | 56 (24) | |
| Surgical abortion by means of curettage (scraping) from the 14th week of pregnancy* *(operativer Abbruch mittels Kürettage (Ausschabung) ab der 14. Woche der Schwangerschaft)* | | | | | | 180 (77.3) | 53 (22.7) | |
| 2. Up to which week of pregnancy is a medical abortion permitted in Germany?*  *(Bis zur wievielten Woche der Schwangerschaft ist in Deutschland ein medikamentöser Schwangerschaftsabbruch erlaubt?)* | Up to the 9th week of pregnancy  *(bis zur 9. Woche der Schwangerschaft)* | | Up to the 12th week of pregnancy  *(bis zur 12. Woche der Schwangerschaft)* | | Up to the 14th week of pregnancy  *(bis zur 14. Woche der Schwangerschaft)* | Up to the 18th week of pregnancy  *(bis zur 18. Woche der Schwangerschaft)* | | Up to the 24th week of pregnancy  *(bis zur 24. Woche der Schwangerschaft)* |
|  | n (%) | | | | | | | |
|  | 65 (29.1) | 118 (52.9) | | 34 (15.2) | | 3 (1.3) | 3 (1.3 | |
| 3. What steps are necessary in Germany before a legal abortion can be carried out?*  *(Welche Schritte sind in Deutschland notwendig, bevor ein legaler Schwangerschaftsabbruch durchgeführt werden kann?)* | | | | | | No / Nein | Yes / Ja | |
|  |  |  |  |  |  | n (%) | | |
| Consultation with a state-certified advice centre* *(Beratungsgespräch durch eine staatlich zertifizierte Beratungsstelle)* | | | | | | 7 (3) | 226 (97) | |
| Waiting time of three days between consultation and termination* *(Wartezeit von drei Tagen zwischen Beratungsgespräch und Abbruch)* | | | | | | 65 (27.9) | 168 (72.1) | |
| Waiting time of two days between consultation and termination* *(Wartezeit von zwei Tagen zwischen Beratungsgespräch und Abbruch)* | | | | | | 182 (78.1) | 51 (21.9) | |
| Foetal heartbeat must be recognizable* *(Herztöne des Fötus müssen erkennbar sein)* | | | | | | 229 (98.3) | 4 (1.7) | |
| Determination of pregnancy by doctor before consultation* *(Feststellung der Schwangerschaft durch Ärzt:in vor Beratungsgespräch)* | | | | | | 50 (21.5) | 183 (78.5) | |
| 4. What percentage of abortions in Germany were performed by medical abortion in 2022?*  *(Wieviel Prozent der Schwangerschaftsabbrüche wurden im Jahr 2022 in Deutschland mittels medikamentöser Methode durchgeführt?)* | 17% of all abortions | | 35% of all abortions | | 52% of all abortions | 69% of all abortions | | 86% of all abortions |
|  | n (%) | | | | | | | |
|  | 31 (14.1) | | 72 (32.7) | | 44 (20.0) | 60 (27.3) | | 13 (5.9) |
| 5. How many practices and clinics performing abortions are currently registered in Germany?*  *(Wie viele Praxen und Kliniken, die Schwangerschaftsabbrüche durchführen, sind in Deutschland aktuell gemeldet?)* | About 500 practices and clinics  *(Ca. 5 00 Praxen und Kliniken)* | | About 1 000 practices and clinics  *(Ca. 1 000 Praxen und Kliniken)* | | About 2 000 practices and clinics  *(Ca. 2 000 Praxen und Kliniken)* | About 3 500 practices and clinics  *(Ca. 3 500 Praxen und Kliniken)* | | About 5 000 practices and clinics  *(Ca. 5 000 Praxen und Kliniken)* |
|  | n (%) | | | | | | | |
|  | 93 (42.3) | | 81 (36.8) | | 33 (15.0) | 11 (5.0) | | 2 (0.9) |
