# Supplementary material for: Who will step up? A cross-sectional study on factors associated with medical students’ willingness to provide abortion care
Source: PLoS One. 2026 Nov 6;21(11):e0362064. doi: 10.1371/journal.pone.0362064 (PMC13742952; doi:10.1371/journal.pone.0362064)
Supplement: Supplementary file 3 [file pone.0362064.s003.docx]

**S3 Appendix.** **Federal states of contacted medical schools and correlations of variables.**

Table A. Distribution of federal states of contacted medical schools in the survey of n = 233 medical students in Germany.

| **Federal state** |  | ***n*** |
| --- | --- | --- |
| Baden-Württemberg |  | 3 |
| Bavaria |  | 6 |
| Berlin |  | 1 |
| Brandenburg |  | 1 |
| Bremen ^a^ |  | 0 |
| Hamburg |  | 1 |
| Hesse |  | 3 |
| Lower Saxony |  | 3 |
| Mecklenburg-West Pomerania |  | 2 |
| North Rhine-Westphalia |  | 9 |
| Rhineland-Palatinate |  | 1 |
| Saarland |  | 1 |
| Saxony |  | 2 |
| Saxony-Anhalt |  | 2 |
| Schleswig-Holstein |  | 2 |
| Thuringia ^b^ |  | 0 |

^a^ There is no university with medical school in Bremen. ^b^ The student council of the medical school did not distribute survey links in general and was thus not contacted.

Table B. Correlations of variables included in the survey of n = 233 medical students in Germany.

| **Variable** | 1. | 2. | 3. | 4. | 5. | 6. | 7. | 8. | 9. | 10. | 11. | 12. | 13. | 14. |
| --- | --- | --- | --- | --- | --- | --- | --- | --- | --- | --- | --- | --- | --- | --- |
| 1. Attitudes towards abortion | — |  |  |  |  |  |  |  |  |  |  |  |  |  |
| 2. Age | .03 | — |  |  |  |  |  |  |  |  |  |  |  |  |
| 3. Gender ^a^ | .32^***^ | -.03 | — |  |  |  |  |  |  |  |  |  |  |  |
| 4. Preferred medical specialisation ^b^ | .01 | .05 | .26^***^ | — |  |  |  |  |  |  |  |  |  |  |
| 5. Influence of faith | -.53^***^ | -.16^*^ | -.12 | -.05 | — |  |  |  |  |  |  |  |  |  |
| 6. Visits of religious sites | -.42^***^ | -.21^**^ | -.08 | -.02 | .75^***^ | — |  |  |  |  |  |  |  |  |
| 7. State of origin ^c^ | .04 | -.03 | .11 | .01 | -.07 | -.06 | — |  |  |  |  |  |  |  |
| 8. Contact | .13* | .27^**^ | .03 | -.00 | -.10 | -.08 | .09 | — |  |  |  |  |  |  |
| 9. Perceived knowledge | -.16^*^ | .27^**^ | -.06 | .05 | -.03 | -.05 | -.07 | .06 | — |  |  |  |  |  |
| 10. Fear of stigmatisation | -.42^***^ | -.00 | -.01 | .05 | .23^**^ | .08 | -.05 | -.09 | -.02 | — |  |  |  |  |
| 11. Perspective-taking | .01 | -.05 | -.04 | .01 | -.01 | .00 | -.05 | -.10 | -.15^*^ | -.03 | — |  |  |  |
| 12. Willingness to participate | .74^***^ | .06 | .31^***^ | .10 | -.36^***^ | -.28^***^ | .08 | .16^*^ | -.09 | -.47^***^ | .03 | — |  |  |
| 13. Subjective norms | .41^***^ | .06 | .10 | -.05 | -.30^***^ | -.19^**^ | -.02 | .11 | -.09 | -.40^***^ | .08 | .39^***^ | — |  |
| 14. Perceived behavioural control | -.21^***^ | -.01 | -.17^***^ | .02 | .12 | .14^*^ | -.06 | -.11 | .09 | .27^***^ | -.05 | -.27^***^ | -.12 | — |

*n* = 223. ^a^ 0 = male, 1 = female. ^b^ 0 = technical, 1 = communicative. ^c^ 0 = Old Federal States, 1 = New Federal States.

^*^*p* < .05. ^**^*p* < .01. ^***^*p* < .001.
