# Supplementary material for: Who will step up? A cross-sectional study on factors associated with medical students’ willingness to provide abortion care
Source: PLoS One. 2026 Nov 6;21(11):e0362064. doi: 10.1371/journal.pone.0362064 (PMC13742952; doi:10.1371/journal.pone.0362064)
Supplement: Supplementary file 4 [file pone.0362064.s005.docx]

PONE-D-26-10064

Who will step up? A cross-sectional study on the determinants of medical students’ willingness to provide abortion care

PLOS One
Javier Fagundo-Rivera, PhD
Academic Editor
==============================

**Journal Requirements:**

When submitting your revision, we need you to address these additional requirements.

1. **Please ensure that your manuscript meets PLOS ONE's style requirements, including those for file naming.** The PLOS ONE style templates can be found at https://journals.plos.org/plosone/s/file?id=wjVg/PLOSOne_formatting_sample_main_body.pdf and https://journals.plos.org/plosone/s/file?id=ba62/PLOSOne_formatting_sample_title_authors_affiliations.pdf

- Thank you for this helpful note. We thoroughly checked the manuscript for PLOS ONE’s style requirements and made the necessary formatting amendments (e.g., table headings and formatting, font sizes, heading formatting, file names).

2. **Please include a new copy of S2 Appendix; the current table is difficult to read.**

- Thank you for pointing this out. We made amendments to table formatting. If you have further suggestions to improve readability, we are very open to make further changes.

**Additional Editor Comments:**

3. Dear Authors,
   Thank you for submitting your manuscript entitled “Who will step up? A cross-sectional study on the determinants of medical students’ willingness to provide abortion care” (Manuscript Number: PONE-D-26-10064) to the journal.
   After evaluation by three independent reviewers and editorial assessment, the current decision is Major Revision.
   The reviewers recognize the potential relevance and originality of the topic. However, they also identified several important issues that should be addressed before the manuscript can be considered further for publication. These concerns include aspects related to methodological clarification, interpretation of findings, statistical reporting, and improvement of the overall presentation and discussion of the results.
   Please revise the manuscript carefully by addressing each reviewer comment point-by-point in a detailed response letter. We encourage you to clearly indicate all modifications made in the revised manuscript, preferably using track changes or highlighted text.
   We look forward to receiving your revised submission and appreciate your contribution to the field.

   Sincerely,

   Javier Fagundo-Rivera, PhD
   Academic Editor

- Thank you very much for considering our manuscript for publication in PLOS One and the opportunity to revise it and strengthen the quality. We appreciate the helpful feedback we received and have carefully revised the manuscript accordingly. Below, we provide our responses to each comment and a description of all corresponding changes we made to the manuscript.

**Reviewers' comments:
Reviewer's Responses to Questions**

**Comments to the Author**

4. Is the manuscript technically sound, and do the data support the conclusions?

   The manuscript must describe a technically sound piece of scientific research with data that supports the conclusions. Experiments must have been conducted rigorously, with appropriate controls, replication, and sample sizes. The conclusions must be drawn appropriately based on the data presented.

Reviewer #1: Yes

Reviewer #2: Yes

Reviewer #3: Yes

5. Has the statistical analysis been performed appropriately and rigorously?

Reviewer #1: Yes

Reviewer #2: Yes

Reviewer #3: Yes

6. **Have the authors made all data underlying the findings in their manuscript fully available?**

   The [PLOS Data policy](http://?) requires authors to make all data underlying the findings described in their manuscript fully available without restriction, with rare exception (please refer to the Data Availability Statement in the manuscript PDF file). The data should be provided as part of the manuscript or its supporting information, or deposited to a public repository. For example, in addition to summary statistics, the data points behind means, medians and variance measures should be available. If there are restrictions on publicly sharing data—e.g. participant privacy or use of data from a third party—those must be specified.

Reviewer #1: Yes

Reviewer #2: No

- Thank you for this important observation. Please see our response to comment no. 17 for our clarification and necessary revision in the manuscript.

Reviewer #3: Yes

7. Is the manuscript presented in an intelligible fashion and written in standard English?

   PLOS ONE does not copyedit accepted manuscripts, so the language in submitted articles must be clear, correct, and unambiguous. Any typographical or grammatical errors should be corrected at revision, so please note any specific errors here.

Reviewer #1: Yes

Reviewer #2: Yes

Reviewer #3: Yes

**Review Comments to the Author**

**Reviewer #1:**

**Major concerns:**

8. **The manuscript overgeneralises findings to “medical students in Germany,” which is not justified.**

- Thank you for highlighting this important issue. We agree that our original wording may have implied a level of generalisability that is not supported by our sampling strategy. To address this concern, we have revised the wording from *“medical students in Germany”* to *“a sample of German medical students”* throughout the whole manuscript*.* This revision should clarify that our findings are based on a non-representative sample and should therefore be interpreted with appropriate caution. In addition, causal and generalising language has been consistently softened throughout the whole manuscript to avoid any overinterpretation.

9. **Clarify handling of missing data. Why ≥10% missing threshold was chosen?**

- Thank you for raising this important methodological point. We have clarified our approach to handling missing data in the revised manuscript and added a corresponding reference (Schafer JL, Graham JW. Missing data: Our view of the state of the art. Psychological Methods. 2002;7(2):147–77. doi: 10.1037/1082-989X.7.2.147.). *“**For multi-item scales, ipsative mean imputation (i.e., replacing a missing value with the mean of the remaining items completed by the same participant) was applied when no more than 10% of the items within a scale were missing [35]. We chose this threshold as a conservative criterion, such that the procedure was only applicable to scales comprising ten or more items.* *Therefore, this threshold was only applied to the scales assessing abortion attitudes (AAS), fear of stigmatisation (APSS) and perspective taking (JSPE). For scales with fewer than ten items, listwise deletion was applied,* *because even a single missing item would exceed the predefined threshold.”*

10. **Ensure consistent use of: “abortion” (not “abortions” in general statements)**

- Thank you for identifying this inconsistency. We carefully reviewed the manuscript and revised the terminology throughout to ensure the consistent use of *“abortion”* in general statements.

11. **The study relies on self-selection via student councils, with: Unknown response rate, Strong gender imbalance (82.8% female). This introduces substantial selection bias.**

- Thank you for this important observation. We agree that our recruitment strategy introduces the possibility of selection bias and have revised the manuscript to address this limitation more explicitly:

To address the unknown response rate, we consistently changed *“German medical students’ attitudes towards abortion”* to *“attitudes towards abortion in a sample of German medical students”* to clarify that we did not survey a representative sample of the population of medical students in Germany. We also expanded the Discussion section to emphasise that participation was voluntary and recruitment was conducted through student councils and possible implications of this approach: *“Additionally, our recruitment strategy through student councils, the voluntary survey participation and the absence of monetary incentives may have led to selective participation, potentially skewing results towards participants with positive attitudes and specific interest in gynaecology.”*

We acknowledge that female students were overrepresented in our sample compared with the current proportion of female medical students in Germany (approximately 65%). We have explicitly acknowledged this limitation in the Discussion section: *“Given that the number of female participants (82.8%) exceeded their proportion within the broader medical student population (64.6%) [34], and that certain federal states were underrepresented, the representativeness of the sample was not achieved.* *The sample was predominantly female with positive attitudes towards abortion, suggesting a particularly engaged and reflective group of participants. This may also help explain why our hypotheses based on the contact hypothesis and the associated mediation effect were not supported. Although participants generally held positive attitudes towards abortion, they reported limited contact with individuals who have had an abortion. The restricted variability in these constructs may also have reduced our ability to detect the hypothesised effects. Consequently, our findings cannot be generalised to the general population of medical students.”*

12. **Clarify whether “subjective knowledge” reflects confidence vs. actual knowledge**

- Thank you for pointing out this ambiguity. As shown in Appendix 2, the scale used in these analyses does not assess actual knowledge (i.e., objectively measured factual knowledge). Instead, it captures participants’ perceived knowledge, including their confidence in their knowledge and their self-reported educational exposure to abortion-related topics. Because no validated instrument measuring abortion-related knowledge among medical students was available, we developed the scale based on items used in previous studies. The items assess whether participants felt sufficiently informed about abortion and whether abortion-related topics to have been covered during their medical education. Objective knowledge (= actual knowledge) was also assessed in the survey; however, this was not part of the present analyses (see Appendix 2). T*o avoid this ambiguity, we* replaced the term *“subjective knowledge”* with *“perceived knowledge”* throughout the manuscript and Appendix 2 and 3, and *“objective knowledge”* with *“factual knowledge”* in Appendix 2. We also have revised the Discussion section to clarify this distinction: *“The measure of perceived knowledge reflects participants' confidence in their knowledge and their perceived educational exposure rather than objective knowledge, making it susceptible to individual differences in self-assessment. For example, students with greater awareness of the legal, ethical, and clinical complexities surrounding abortion may rate their own knowledge more critically than students with more limited insight, resulting in lower perceived knowledge despite greater actual understanding. Conversely, less informed participants may overestimate their knowledge [43].”*

13. **The negative association between: subjective knowledge and attitudes, and perceived behavioural control and willingness contradicts theory, yet is insufficiently explained. This suggests: Possible measurement issues Or misinterpretation of the construct.......Recommendation: Provide theoretical explanation or acknowledge inconsistency more explicitly**

- Thank you for this thoughtful observation. We agree that these findings require further discussion. We have therefore expanded the Discussion: *“The negative associations were unexpected and are* *not fully consistent with the assumptions of intergroup contact theory and the theory of planned behaviour.* *Several explanations are possible. The measure of subjective knowledge reflects participants' perceived rather than objective knowledge, making it susceptible to individual differences in self-assessment. For example, students with greater awareness of the legal, ethical, and clinical complexities surrounding abortion may rate their own knowledge more critically than students with more limited insight, resulting in lower perceived knowledge despite greater actual understanding. Conversely, less informed participants may overestimate their knowledge [43]. The construct of perceived behavioural control in our study may have captured perceived external barriers to providing abortion care, such as legal restrictions, limited training opportunities, institutional barriers, or anticipated stigma, rather than confidence in one's own abilities alone. Consequently, students who had reflected more extensively on the practical realities of abortion provision may have reported lower perceived behavioural control despite expressing generally favourable attitudes toward abortion.”* We therefore changed *“subjective knowledge”* to *“perceived knowledge”* for clarification (see our response to comment no. 11). Because our study was cross-sectional, our findings should be interpreted cautiously and do not permit causal conclusions, we have added these considerations to the Discussion and explicitly acknowledge that future studies should further examine the validity of these constructs*: “The survey utilised a combination of established scales and self-developed items due to the lack of validated instruments, resulting in variable internal consistency. Thus, scales assessing constructs such as contact, perceived knowledge, and perceived behavioural control require further validation in future studies. Results that relate to these constructs should therefore be interpreted with appropriate caution.” and “The cross-sectional design limits the ability to infer causality from the observed associations.”*

14. **Needs language polishing like
    Abstract:
    - factors contributing to explain medical students' attitudes may be written as “factors associated with medical students’ attitudes”
    - Fear of stigmatisation and subjective knowledge were identified as significant negative predictors may be written as “Fear of stigmatisation and subjective knowledge were negatively associated with attitudes”**

    **Introduction:
    - Internationally, recent years have seen a reversal of legislative liberalisation...may be written as “Internationally, recent years have seen shifts in abortion legislation, including both liberalisation and restriction…”
    - Research consistently shows that restricting abortion access does not reduce the number of abortions...may be written as “Previous research suggests that restricting abortion access does not necessarily reduce the number of abortions…”**

    **Methods:
    - items deleted to increase reliability if below .60 as “items were removed if necessary to achieve acceptable internal consistency (α ≥ .60)”**

    **Results:**
    **- showed no association as “was not statistically associated”**

    **Discussion:
    - could hinder young physicians as “may represent barriers for future physicians”**
    **- confirms a strong positive association as “is consistent with previous findings showing a strong positive association” (Avoids overclaiming)**

- *Thank you for the helpful feedback to improve the language of our manuscript. We revised the manuscript accordingly.*

**Reviewer #2:**

15. **The language used throughout the article is "predicted" and it reflects causality. This is something that cannot be infered from a survey administered in a single data point. Please soften the tone throughout the article and on the title too.**

- *Thank you for this important comment. We agree that causal language is not appropriate given the cross-sectional design of our study. We have softened the tone throughout the manuscript (as also advised in reviewer comment no. 14) and have revised the title and manuscript by changing “determinants of” to “factors associated with” and “predicted” to “was associated with”.*

16. **Attitudes correlate with willingness at r = .74, and the willingness items are themselves substantially attitudinal. Please discuss in the discussion if there is a risk of conceptual overlap rather than a meaningful predictor-outcome relationship.**

- Thank you for highlighting this important aspect. We agree that there may indeed be a conceptual overlap between the constructs and have added this in the Discussion section: *“**The strong association between abortion attitudes and willingness to provide abortion care may, in part, reflect conceptual overlap between the measures. While the AAS assesses general attitudes about abortion, willingness to provide abortion care reflects the behavioural expression of these attitudes. Although willingness constitutes a distinct construct by assessing professional behavioural intentions, it is theoretically expected to be strongly influenced by underlying attitudes toward abortion [21].”*

17. **Fix the data availability statement. The manuscript says data are available from the corresponding author on request. The submission form says they are on OSF without restriction.**

- Thank you for pointing this out. We have corrected the Data Availability Statement to ensure consistency with the submission information. The revised statement now indicates that the data are openly available on OSF without restriction: “*The datasets used and/or analysed during the current study are available on OSF (https://osf.io/zbxah/) with no restriction.”*

18. **Reference 28 is described as a secondary analysis of a cross-sectional study among medical students, and you reference the CarePreg project. This looks like the same dataset feeding more than one paper. Please declare openly**

- Thank you for this note. You are correct that the secondary analysis reported in reference 28 (now 31 in the revised manuscript) was conducted using the same dataset as the present study. To make this more transparent, we revised the relevant paragraph to *“The AAS had to be adapted explicitly for the German cultural and political context of the present. Thus, after data collection for the study at hand, we conducted a secondary analysis to assess psychometric properties of the AAS using the data set collected in the present study with medical students [31].”*

19. **Foreground the null mediation result. The contact pathway through knowledge, perspective-taking, and fear of stigmatisation did not hold, and the direct contact effect was non-significant. Your introduction builds this up at length, so the abstract and discussion need to state clearly that it did not pan out.**

- Thank you for this helpful suggestion. We agree that the null findings should be more prominently reported. Accordingly, we have revised both the Abstract *(“The association between attitudes and contact, as well as the expected mediation effects of fear of stigmatisation, perceived knowledge and perspective-taking, were nonsignificant.“)* and the Discussion *(“The expected indirect mediation effect between attitudes and contact through knowledge, perspective-taking and fear of stigmatisation was also not confirmed in our sample.“)* to clearly state that the hypothesised indirect pathway through knowledge, perspective-taking, and fear of stigmatisation was not supported and that the direct effect of contact was also non-significant. We also discuss the implications of these findings with regard to the selection bias in our sample. *“The study sample was predominantly female with positive attitudes towards abortion, suggesting a particularly engaged and reflective group of participants. This may also help explain why our hypotheses based on the contact hypothesis and the associated mediation effect were not supported. Although participants generally held positive attitudes towards abortion, they reported limited contact with individuals who have had an abortion. The restricted variability in these constructs may also have reduced our ability to detect the hypothesised effects. Consequently, our findings cannot be generalised to the general population of medical students.“*

**Reviewer #3:**

20. **The manuscript by Mirja Baumgart et.al is a statistical, descriptive study performed in a sample of medicine students in Germany, focused in describe attitudes and, factors influencing these attitudes, toward abortion. The idea is good, attractive and pretends to influence in the country’s general context regarding acceptance of this procedure, with a slightly covered pretension to influence on medicine schools’ curricula. There are no methodological flaws, and results support conclusions. The manuscript is well-written and has minimal observations. English language is acceptable.**

- Thank you for your kind feedback and your helpful comments. We are happy for each opportunity to improve our manuscript.

I have only a few observations:

**Methodology**

21. **Medicine schools in Germany are private or have government participation? In the latter, what proportion of students participate?**

- Thank you for this important question. In Germany, the majority of medical schools are public universities funded by the government. Approximately 10% of all medical students in Germany are enrolled at private medical schools. However, both types (public and private universities) follow the same binding, nationwide regulations governing medical studies (“Approbationsordnung”). To clarify this, we have added information on the status of the contacted medicals schools to the Methods section under the subsection Data collection. We also corrected a minor error regarding the total number of medical schools that were contacted from 38 to 37: *“For this purpose, these institutions were first identified, and a list of all 42 German medical schools (36 public, 6 private) at the time with medical student councils and their email addresses was compiled. Five medical schools either did not provide contact details (n = 4) or stated directly that they do not distribute survey links (n = 1) and were thus not contacted. Medical school student councils of 37 medical schools (35 public, 2 private) were contacted between September 8th and 18th, 2023 via email and asked to distribute the survey link via mailing lists, social media and digital bulletin boards.”*

22. **Students were divided according to the grade they were enrolled in? A student from the first semester is different to another in 13^th^ semester.**

- Thank you for raising this important point. We did not include semester of study as a predictor because abortion was not part of the standard medical curriculum in Germany at the time of data collection. Consequently, exposure to abortion-related education was expected to depend more on individual learning experiences than on curricular progression. Therefore, we assumed that students’ decision to seek information about abortion were independent of their semester of study. However, we included age as a predictor, as it is strongly correlated with the stage of medical training, and it did not correlate with abortion attitudes in our analyses.

23. **Row 157 in methodology: What is Mage? Please clarify.**

- Thank you for identifying this formatting issue. We have revised the text to *“age: M = 24.80 years”* to clarify that this refers to the mean age of the students.

**Results:**

24. **How many medicine schools participated? Where were they located?**

- Thank you for this valuable suggestion. As stated in the Methods section, 37 medical schools were contacted for participation. These institutions were located across Germany. However, we did not record the distribution of participants by individual medical school, but only by federal state. It is therefore impossible to draw any conclusions regarding the exact number of participating medical schools. To improve transparency, we have expanded the description of the medical schools and their geographical distribution in the Methods section of the revised manuscript: *“Medical school student councils of 37 medical schools (35 public, 2 private) were contacted between September 8th and 18th, 2023 via email and asked to distribute the survey link via mailing lists, social media and digital bulletin boards. A table showing the distribution of contacted medical schools per federal state can be found in S3 Appendix. In the absence of any feedback regarding the process of link distribution, it is not possible to draw any inferences regarding the number of students who were actually reached or the medical schools, which were represented in our sample.”*

**Additional changes made to the manuscript:**

In June 2026, a cross-sectional study with 213 German gynaecologists and other abortion providers of all specialties by Le et al. was published (Le V, Christalle E, Rutenkröger M, Zill J, Scholl I, Lindig, A. Barriers and motivations: analyzing the factors influencing abortion provision by gynecologists in Germany – a cross-sectional study. Reprod Health. 2026;23(1):125. doi: 10.1186/s12978-026-02389-8.). We added this important information in the Introduction section: *“Despite the ongoing discourse surrounding abortion law and education, to date only one quantitative and few qualitative studies have explored the attitudes of medical students and physicians towards abortion in Germany [10, 13, 26]. In addition to the above-mentioned study by Lindig et al. [13], Baier and Behnke [10] conducted an interview study with 14 medical students and four physicians and revealed barriers for specialisation and participation in abortion care including misinformation, lack of knowledge, fear of stigmatisation, and religious beliefs. In a cross-sectional study drawing on the theory of planned behaviour by Le et al. with 213 gynaecologists and abortion providers of all specialties, perceived behavioural control was the strongest predictor of intention to provide abortion care, followed by subjective norms and attitudes towards abortion. Abortion providers reported significantly more positive attitudes, stronger subjective norms, higher perceived behavioural control, lower fear of stigmatisation and higher subjective knowledge of abortion care in comparison to non-providers [26].”*

We also discussed these relevant results with regard to our results in the Discussion section: *“However, the results contrast with Le et al.’s findings who reported that perceived behavioural control was the strongest predictor of behavioural intention to provide abortion care, with attitudes being the weakest predictor [26]. One possible explanation might be the difference in the study populations. Le et al. surveyed practising physicians, most of whom were between the age of 40 and 59 and had substantial professional experience. In contrast, our sample consisted of students with little or no clinical experience. Without relevant professional experience, students may rely more on their personal attitudes when forming behavioural intentions, whereas for practising physicians, intentions may be influenced more strongly by perceived behavioural control, reflecting their confidence in performing the behaviour, and by subjective norms related to their professional environment.”*
