# Supplementary material for: Who will step up? A cross-sectional study on factors associated with medical students’ willingness to provide abortion care
Source: PLoS One. 2026 Nov 6;21(11):e0362064. doi: 10.1371/journal.pone.0362064 (PMC13742952; doi:10.1371/journal.pone.0362064)
Supplement: Supplementary file 5 [file pone.0362064.s006.docx]

PONE-D-26-10064R1
Who will step up? A cross-sectional study on factors associated with medical students’ willingness to provide abortion care
PLOS One

Helen Howard
Staff Editor

**Journal Requirements:**

If the reviewer comments include a recommendation to cite specific previously published works, please review and evaluate these publications to determine whether they are relevant and should be cited. There is no requirement to cite these works unless the editor has indicated otherwise.

Please review your reference list to ensure that it is complete and correct. If you have cited papers that have been retracted, please include the rationale for doing so in the manuscript text, or remove these references and replace them with relevant current references. Any changes to the reference list should be mentioned in the rebuttal letter that accompanies your revised manuscript. If you need to cite a retracted article, indicate the article’s retracted status in the References list and also include a citation and full reference for the retraction notice.

- Thank you and the reviewers for the opportunity to further revise and strengthen our manuscript. We appreciate the constructive feedback provided throughout the review process and hope that the revisions made in this minor revision have addressed all remaining concerns and that the manuscript now fully meets PLOS One’s publication criteria.

The reviewers did not recommend any additional references to be cited. We nevertheless carefully checked the reference list and did not identify any further amendments that were necessary. Below, we address the points raised by the reviewers, along with quotations of the corresponding revised text.

**Reviewers' comments:**

**Review Comments to the Author**

**Reviewer #1:** Thank you for the careful revision of the manuscript. The authors have addressed most of the major concerns raised in the previous review, and the revised manuscript is substantially improved. In particular, the authors have appropriately moderated the generalizability of the findings, clarified the recruitment strategy and potential selection bias, and provided a more explicit discussion.
I consider the manuscript suitable for publication after some minor revisions:

- Thank you for your positive and constructive feedback. We appreciate the recognition of the improvements made and have carefully addressed the remaining minor comments.

**1. Missing data: The procedure for handling missing data is now clearly described. However, the rationale for selecting the 10% threshold for item-level imputation could be explained more explicitly, preferably with a methodological reference supporting this choice.**

- Thank you for this helpful suggestion. We agree that the rationale for the 10% threshold should be explained more explicitly. The threshold was not selected on the basis of a specific published cut-off, but was determined a priori through discussion within the research team as a pragmatic and conservative criterion for limiting item-level imputation. Our rational was that imputation should only be applied when the amount of missing information within a scale was very small. The 10% threshold was considered to be sufficiently conservative for this purpose.

We have revised the Methods section to make this rationale explicit and to clarify that the 10% threshold represents a study-specific, pragmatic criterion rather than a universally established methodological cut-off: *“For multi-item scales, person-level mean imputation within a scale (i.e., replacing a missing value with the mean of the remaining items completed by the same participant) [35] was applied when no more than 10% of the items within a scale were missing. This threshold was applied a priori as a pragmatic criterion, ensuring that imputation was only performed when the proportion of missing item information was small. Thus, imputation was only applicable to scales comprising at least ten items. Therefore, this imputation was only applied to the scales assessing abortion attitudes (AAS, 14 items), fear of stigmatisation (APSS, 10 items) and perspective taking (JSPE, 10 items),* *For scales containing less than ten items or in case of missing single items, even a single missing item would exceed the predefined threshold and participants with missing item responses were excluded from the respective scale analysis.”*

**2. Minor language editing: A final proofreading for grammatical consistency and terminology would improve readability. For example, please ensure consistent use of “abortion,” “perceived knowledge,” “perceived behavioural control,” and “willingness to participate in abortion care.”**

- Thank you for this suggestion. We have carefully proofread the entire manuscript and corrected grammatical inconsistencies, terminology and wording where necessary. We have also ensured consistent use of the terms “abortion,” “perceived knowledge,” “perceived behavioural control,” and “willingness to participate in abortion care” throughout the manuscript, e.g., we changed *“willingness to provide abortions”* into *“willingness to participate in abortion care”* and *“abortion attitudes”* into *“attitudes towards abortion”*.

**3. Overall, the revision has responded well to the substantive concerns raised previously. I therefore recommend minor revision.**

- Thank you for your positive assessment and constructive feedback. We have addressed all of the remaining minor issued raised in this review and believe that these revisions have further improved the clarity and consistency of the manuscript.

**Reviewer #2:** I think the article is ready for acceptance. The authors have addressed the reviewers concerns adequately. Three super small last things to check:

- Thank you for your positive assessment of the manuscript and for confirming that the previous concerns have been adequately addressed. Thank you very much for checking the manuscript so thoroughly. We have carefully checked and corrected each of the three points identified.

**1. The text reports n = 192 female participants (82.8%) but Table 2 says 193. The table is probably correct.**

- Thank you for identifying this inconsistency. You are correct, the number of female participants is 193, as reported in Table 2. We have corrected the corresponding value in the main text to ensure consistency: *“most participants were female (n = 193, 82.8%).”*

**2. "Fifteen participants (0.04%) did not answer a single question" should be 4.3%. That's a proportion reported as a percentage.**

- Thank you for catching this error. Indeed, the 0.04 is the decimal value and not the percentage. The correct percentage is 4.3%. We have corrected it to *“Fifteen participants (4.3%) did not answer a single question”*.

**3. Text says 40.5% never visited religious sites; Table 2 correctly says 40.3%.**

- Thank you for identifying this discrepancy. The correct percentage is 40.3%, consistent with Table 2. We have corrected it in the main text accordingly: *“However, 152 participants (65.2%) stated that they were not influenced by religious beliefs, and 94 (40.3%) never visited religious sites.”*

**Reviewer #3:** The previous comments have been considered by the authors and the manuscript modified accordingly. I have no more comments

- Thank you for your positive evaluation of our manuscript and for confirming that the previous comments have been adequately addressed.
