# Supplementary material for: Who will step up? A cross-sectional study on factors associated with medical students’ willingness to provide abortion care
Source: PLoS One. 2026 Nov 6;21(11):e0362064. doi: 10.1371/journal.pone.0362064 (PMC13742952; doi:10.1371/journal.pone.0362064)
Supplement: Peer review history

# Peer review: Decision Letter 0

**DOI:** 10.1371/journal.pone.0362064.r001

**Authors:** Javier Fagundo-Rivera

19 Jun 2026

PONE-D-26-10064

Who will step up? A cross-sectional study on the determinants of medical students’ willingness to provide abortion care

PLOS One?

Dear Dr. Lindig,

Thank you for submitting your manuscript to PLOS ONE. After careful consideration, we feel that it has merit but does not fully meet PLOS ONE’s publication criteria as it currently stands. Therefore, we invite you to submit a revised version of the manuscript that addresses the points raised during the review process.

==============================

**ACADEMIC EDITOR:**

Dear Authors,

Thank you for submitting your manuscript entitled “Who will step up? A cross-sectional study on the determinants of medical students’ willingness to provide abortion care” (Manuscript Number: PONE-D-26-10064) to the journal.

After evaluation by three independent reviewers and editorial assessment, the current decision is Major Revision.

The reviewers recognize the potential relevance and originality of the topic. However, they also identified several important issues that should be addressed before the manuscript can be considered further for publication. These concerns include aspects related to methodological clarification, interpretation of findings, statistical reporting, and improvement of the overall presentation and discussion of the results.

Please revise the manuscript carefully by addressing each reviewer comment point-by-point in a detailed response letter. We encourage you to clearly indicate all modifications made in the revised manuscript, preferably using track changes or highlighted text.

We look forward to receiving your revised submission and appreciate your contribution to the field.

Sincerely,

Javier Fagundo-Rivera, PhD

Academic Editor

==============================

Please submit your revised manuscript by Aug 03 2026 11:59PM. If you will need more time than this to complete your revisions, please reply to this message or contact the journal office at plosone@plos.org. When you're ready to submit your revision, log on to https://www.editorialmanager.com/pone/ and select the 'Submissions Needing Revision' folder to locate your manuscript file.

- A letter that responds to each point raised by the academic editor and reviewer(s). You should upload this letter as a separate file labeled 'Response to Reviewers'.
- A marked-up copy of your manuscript that highlights changes made to the original version. You should upload this as a separate file labeled 'Revised Manuscript with Track Changes'.
- An unmarked version of your revised paper without tracked changes. You should upload this as a separate file labeled 'Manuscript'.

If you would like to make changes to your financial disclosure, please include your updated statement in your cover letter. Guidelines for resubmitting your figure files are available below the reviewer comments at the end of this letter.

If applicable, we recommend that you deposit your laboratory protocols in protocols.io to enhance the reproducibility of your results. Protocols.io assigns your protocol its own identifier (DOI) so that it can be cited independently in the future. For instructions see: https://journals.plos.org/plosone/s/submission-guidelines#loc-laboratory-protocols. Additionally, PLOS ONE offers an option for publishing peer-reviewed Lab Protocol articles, which describe protocols hosted on protocols.io. Read more information on sharing protocols at https://plos.org/protocols?utm_medium=editorial-email&utm_source=authorletters&utm_campaign=protocols.

As the corresponding author, your ORCID iD is verified in the submission system and will appear in the published article. PLOS supports the use of ORCID, and we encourage all coauthors to register for an ORCID iD and use it as well. Please encourage your coauthors to verify their ORCID iD within the submission system before final acceptance, as unverified ORCID iDs will not appear in the published article. *Only*  the individual author can complete the verification step; PLOS staff *cannot*  verify ORCID iDs on behalf of authors.

We look forward to receiving your revised manuscript.

Kind regards,

Javier Fagundo-Rivera, PhD

Academic Editor

PLOS One

Journal Requirements:

When submitting your revision, we need you to address these additional requirements.

1. Please ensure that your manuscript meets PLOS ONE's style requirements, including those for file naming. The PLOS ONE style templates can be found at https://journals.plos.org/plosone/s/file?id=wjVg/PLOSOne_formatting_sample_main_body.pdf and https://journals.plos.org/plosone/s/file?id=ba62/PLOSOne_formatting_sample_title_authors_affiliations.pdf

2. Please include a new copy of S2 Appendix; the current table is difficult to read.

If the reviewer comments include a recommendation to cite specific previously published works, please review and evaluate these publications to determine whether they are relevant and should be cited. There is no requirement to cite these works unless the editor has indicated otherwise.

**Additional Editor Comments:**

Dear Authors,

Thank you for submitting your manuscript entitled “Who will step up? A cross-sectional study on the determinants of medical students’ willingness to provide abortion care” (Manuscript Number: PONE-D-26-10064) to the journal.

After evaluation by three independent reviewers and editorial assessment, the current decision is Major Revision.

The reviewers recognize the potential relevance and originality of the topic. However, they also identified several important issues that should be addressed before the manuscript can be considered further for publication. These concerns include aspects related to methodological clarification, interpretation of findings, statistical reporting, and improvement of the overall presentation and discussion of the results.

Please revise the manuscript carefully by addressing each reviewer comment point-by-point in a detailed response letter. We encourage you to clearly indicate all modifications made in the revised manuscript, preferably using track changes or highlighted text.

We look forward to receiving your revised submission and appreciate your contribution to the field.

Sincerely,

Javier Fagundo-Rivera, PhD

Academic Editor

[Note: HTML markup is below. Please do not edit.]

Reviewers' comments:

Reviewer's Responses to Questions

**Comments to the Author**

1. Is the manuscript technically sound, and do the data support the conclusions?

Reviewer #1: Yes

Reviewer #2: Yes

Reviewer #3: Yes

**********

2. Has the statistical analysis been performed appropriately and rigorously?

Reviewer #1: Yes

Reviewer #2: Yes

Reviewer #3: Yes

**********

3. Have the authors made all data underlying the findings in their manuscript fully available?

The PLOS Data policy

Reviewer #1: Yes

Reviewer #2: No

Reviewer #3: Yes

**********

4. Is the manuscript presented in an intelligible fashion and written in standard English?

Reviewer #1: Yes

Reviewer #2: Yes

Reviewer #3: Yes

**********

**Reviewer #1:**

Major concerns:

- The manuscript overgeneralises findings to “medical students in Germany,” which is not justified.

- Clarify handling of missing data. Why ≥10% missing threshold was chosen?

- Ensure consistent use of: “abortion” (not “abortions” in general statements)

- The study relies on self-selection via student councils, with: Unknown response rate, Strong gender imbalance (82.8% female). This introduces substantial selection bias.

- Clarify whether “subjective knowledge” reflects confidence vs. actual knowledge

- The negative association between: subjective knowledge and attitudes, and perceived behavioural control and willingness contradicts theory, yet is insufficiently explained. This suggests: Possible measurement issues Or misinterpretation of the construct.......Recommendation: Provide theoretical explanation or acknowledge inconsistency more explicitly

Needs language polishing like

Abstract:

- factors contributing to explain medical students' attitudes may be written as “factors associated with medical students’ attitudes”

- Fear of stigmatisation and subjective knowledge were identified as significant negative predictors may be written as “Fear of stigmatisation and subjective knowledge were negatively associated with attitudes”

Introduction:

- Internationally, recent years have seen a reversal of legislative liberalisation...may be written as “Internationally, recent years have seen shifts in abortion legislation, including both liberalisation and restriction…”

- Research consistently shows that restricting abortion access does not reduce the number of abortions...may be written as “Previous research suggests that restricting abortion access does not necessarily reduce the number of abortions…”

Methods:

- items deleted to increase reliability if below .60 as “items were removed if necessary to achieve acceptable internal consistency (α ≥ .60)”

Results:

- showed no association as “was not statistically associated”

Discussion:

- could hinder young physicians as “may represent barriers for future physicians”

- confirms a strong positive association as “is consistent with previous findings showing a strong positive association” (Avoids overclaiming)

**Reviewer #2:**

-The language used throughout the article is "predicted" and it reflects causality. This is something that cannot be infered from a survey administered in a single data point. Please soften the tone throughout the article and on the title too.

-Attitudes correlate with willingness at r = .74, and the willingness items are themselves substantially attitudinal. Please discuss in the discussion if there is a risk of conceptual overlap rather than a meaningful predictor-outcome relationship.

-Fix the data availability statement. The manuscript says data are available from the corresponding author on request. The submission form says they are on OSF without restriction.

Reference 28 is described as a secondary analysis of a cross-sectional study among medical students, and you reference the CarePreg project. This looks like the same dataset feeding more than one paper. Please declare openly

Foreground the null mediation result. The contact pathway through knowledge, perspective-taking, and fear of stigmatisation did not hold, and the direct contact effect was non-significant. Your introduction builds this up at length, so the abstract and discussion need to state clearly that it did not pan out.

**Reviewer #3:**

The manuscript by Mirja Baumgart et.al is a statistical, descriptive study performed in a sample of medicine students in Germany, focused in describe attitudes and, factors influencing these attitudes, toward abortion. The idea is good, attractive and pretends to influence in the country’s general context regarding acceptance of this procedure, with a slightly covered pretension to influence on medicine schools’ curricula. There are no methodological flaws, and results support conclusions. The manuscript is well-written and has minimal observations. English language is acceptable.

I have only a few observations:

Methodology

-Medicine schools in Germany are private or have government participation? In the latter, what proportion of students participate?

- Students were divided according to the grade they were enrolled in? A student from the first semester is different to another in 13th semester.

-Row 157 in methodology: What is Mage? Please clarify.

Results:

How many medicine schools participated? Where were they located?

**********

what does this mean?). If published, this will include your full peer review and any attached files.

If you choose “no”, your identity will remain anonymous but your review may still be made public.

**Do you want your identity to be public for this peer review?**  For information about this choice, including consent withdrawal, please see our Privacy Policy

Reviewer #1: No

Reviewer #2: **Yes:**  Francisco Javier Ruiloba Portilla

Reviewer #3: No

**********

[NOTE: If reviewer comments were submitted as an attachment file, they will be attached to this email and accessible via the submission site. Please log into your account, locate the manuscript record, and check for the action link "View Attachments". If this link does not appear, there are no attachment files.]

To ensure your figures meet our technical requirements, please review our figure guidelines: https://journals.plos.org/plosone/s/figures

You may also use PLOS’s free figure tool, NAAS, to help you prepare publication quality figures: https://journals.plos.org/plosone/s/figures#loc-tools-for-figure-preparation.

NAAS will assess whether your figures meet our technical requirements by comparing each figure against our figure specifications.

# Author comment: Author response to Decision Letter 1

**DOI:** 10.1371/journal.pone.0362064.r002

2 Aug 2026

For a detailed response to the editors and reviewers comments, please see the uploaded document "responese letter".

Attachment Submitted filename: Baumgart et al_Major Revision_Responseletter.docx

# Peer review: Decision Letter 1

**DOI:** 10.1371/journal.pone.0362064.r003

**Authors:** Helen Howard

4 Sep 2026

Who will step up? A cross-sectional study on factors associated with medical students’ willingness to provide abortion care

PLOS One

Dear Dr. Lindig,

Thank you for submitting your manuscript to PLOS One. After careful consideration, we feel that it has merit but does not fully meet PLOS One’s publication criteria as it currently stands. Therefore, we invite you to submit a revised version of the manuscript that addresses the points raised during the review process.

Please submit your revised manuscript by Oct 18 2026 11:59PM. If you will need more time than this to complete your revisions, please reply to this message or contact the journal office at plosone@plos.org. When you're ready to submit your revision, log on to https://www.editorialmanager.com/pone/ and select the 'Submissions Needing Revision' folder to locate your manuscript file.

- A letter that responds to each point raised by the academic editor and reviewer(s). You should upload this letter as a separate file labeled 'Response to Reviewers'.
- A marked-up copy of your manuscript that highlights changes made to the original version. You should upload this as a separate file labeled 'Revised Manuscript with Track Changes'.
- An unmarked version of your revised paper without tracked changes. You should upload this as a separate file labeled 'Manuscript'.

If you would like to make changes to your financial disclosure, please include your updated statement in your cover letter. Guidelines for resubmitting your figure files are available below the reviewer comments at the end of this letter.

If applicable, we recommend that you deposit your laboratory protocols in protocols.io to enhance the reproducibility of your results. Protocols.io assigns your protocol its own identifier (DOI) so that it can be cited independently in the future. For instructions see: https://journals.plos.org/plosone/s/submission-guidelines#loc-laboratory-protocols. Additionally, PLOS One offers an option for publishing peer-reviewed Lab Protocol articles, which describe protocols hosted on protocols.io. Read more information on sharing protocols at https://plos.org/protocols?utm_medium=editorial-email&utm_source=authorletters&utm_campaign=protocols.

As the corresponding author, your ORCID iD is verified in the submission system and will appear in the published article. PLOS supports the use of ORCID, and we encourage all coauthors to register for an ORCID iD and use it as well. Please encourage your coauthors to verify their ORCID iD within the submission system before final acceptance, as unverified ORCID iDs will not appear in the published article. *Only*  the individual author can complete the verification step; PLOS staff *cannot*  verify ORCID iDs on behalf of authors.

We look forward to receiving your revised manuscript.

Kind regards,

Helen Howard

Staff Editor

PLOS One

Journal Requirements:

If the reviewer comments include a recommendation to cite specific previously published works, please review and evaluate these publications to determine whether they are relevant and should be cited. There is no requirement to cite these works unless the editor has indicated otherwise.

Please review your reference list to ensure that it is complete and correct. If you have cited papers that have been retracted, please include the rationale for doing so in the manuscript text, or remove these references and replace them with relevant current references. Any changes to the reference list should be mentioned in the rebuttal letter that accompanies your revised manuscript. If you need to cite a retracted article, indicate the article’s retracted status in the References list and also include a citation and full reference for the retraction notice.

[Note: HTML markup is below. Please do not edit.]

Reviewers' comments:

Reviewer's Responses to Questions

**Comments to the Author**

Reviewer #1: All comments have been addressed

Reviewer #2: All comments have been addressed

Reviewer #3: All comments have been addressed

**********

2. Is the manuscript technically sound, and do the data support the conclusions?

Reviewer #1: Yes

Reviewer #2: Yes

Reviewer #3: (No Response)

**********

3. Has the statistical analysis been performed appropriately and rigorously?

Reviewer #1: Yes

Reviewer #2: Yes

Reviewer #3: (No Response)

**********

4. Have the authors made all data underlying the findings in their manuscript fully available?

The PLOS Data policy

Reviewer #1: Yes

Reviewer #2: Yes

Reviewer #3: (No Response)

**********

5. Is the manuscript presented in an intelligible fashion and written in standard English?

Reviewer #1: Yes

Reviewer #2: Yes

Reviewer #3: (No Response)

**********

Reviewer #1: Thank you for the careful revision of the manuscript. The authors have addressed most of the major concerns raised in the previous review, and the revised manuscript is substantially improved. In particular, the authors have appropriately moderated the generalizability of the findings, clarified the recruitment strategy and potential selection bias, and provided a more explicit discussion

I consider the manuscript suitable for publication after some minor revisions:

1. Missing data: The procedure for handling missing data is now clearly described. However, the rationale for selecting the 10% threshold for item-level imputation could be explained more explicitly, preferably with a methodological reference supporting this choice.

2. Minor language editing: A final proofreading for grammatical consistency and terminology would improve readability. For example, please ensure consistent use of “abortion,” “perceived knowledge,” “perceived behavioural control,” and “willingness to participate in abortion care.”

Overall, the revision has responded well to the substantive concerns raised previously. I therefore recommend minor revision.

Reviewer #2: I think the article is ready for acceptance. The authors have addressed the reviewers concerns adequately. Three super small last things to check:

The text reports n = 192 female participants (82.8%) but Table 2 says 193. The table is probably correct.

"Fifteen participants (0.04%) did not answer a single question" should be 4.3%. That's a proportion reported as a percentage.

Text says 40.5% never visited religious sites; Table 2 correctly says 40.3%.

Reviewer #3: The previous comments have been considered by the authors and the manuscript modified accordingly. I have no more comments

**********

what does this mean?). If published, this will include your full peer review and any attached files.

If you choose “no”, your identity will remain anonymous but your review may still be made public.

**Do you want your identity to be public for this peer review?**  For information about this choice, including consent withdrawal, please see our Privacy Policy

Reviewer #1: No

Reviewer #2: **Yes:**  Francisco Javier Ruiloba Portilla

Reviewer #3: No

**********

[NOTE: If reviewer comments were submitted as an attachment file, they will be attached to this email and accessible via the submission site. Please log into your account, locate the manuscript record, and check for the action link "View Attachments". If this link does not appear, there are no attachment files.]

To ensure your figures meet our technical requirements, please review our figure guidelines: https://journals.plos.org/plosone/s/figures

You may also use PLOS’s free figure tool, NAAS, to help you prepare publication quality figures: https://journals.plos.org/plosone/s/figures#loc-tools-for-figure-preparation.

NAAS will assess whether your figures meet our technical requirements by comparing each figure against our figure specifications.

# Author comment: Author response to Decision Letter 2

**DOI:** 10.1371/journal.pone.0362064.r004

4 Oct 2026

Thank you very much for the opportunity to again revise our manuscript. For a detailed overview of the revised sections and our comments to the reviewers suggestions, please the the document "Response to reviewers" and the manuscript with tracked changes in the attached files section.

Attachment Submitted filename: Baumgart et al_Response to Reviewers_minor_revision.docx

# Peer review: Decision Letter 2

**DOI:** 10.1371/journal.pone.0362064.r005

**Authors:** Andrea Cioffi

21 Oct 2026

Who will step up? A cross-sectional study on factors associated with medical students’ willingness to provide abortion care

PONE-D-26-10064R2

Dear Dr. Lindig,

We’re pleased to inform you that your manuscript has been judged scientifically suitable for publication and will be formally accepted for publication once it meets all outstanding technical requirements.

Within one week, you’ll receive an e-mail detailing the required amendments. When these have been addressed, you’ll receive a formal acceptance letter and your manuscript will be scheduled for publication.

An invoice will be generated when your article is formally accepted. Please note, if your institution has a publishing partnership with PLOS and your article meets the relevant criteria, all or part of your publication costs will be covered. Please make sure your user information is up-to-date by logging into Editorial Manager at Editorial Manager® and clicking the ‘Update My Information' link at the top of the page. For questions related to billing, please contact billing support.

If your institution or institutions have a press office, please notify them about your upcoming paper to help maximize its impact. If they’ll be preparing press materials, please inform our press team as soon as possible -- no later than 48 hours after receiving the formal acceptance. Your manuscript will remain under strict press embargo until 2 pm Eastern Time on the date of publication. For more information, please contact onepress@plos.org.

Kind regards,

Andrea Cioffi

Academic Editor

PLOS One

Additional Editor Comments (optional):

Reviewers' comments:

Reviewer's Responses to Questions

**Comments to the Author**

Reviewer #3: All comments have been addressed

**********

2. Is the manuscript technically sound, and do the data support the conclusions?

Reviewer #3: (No Response)

**********

3. Has the statistical analysis been performed appropriately and rigorously?

Reviewer #3: (No Response)

**********

4. Have the authors made all data underlying the findings in their manuscript fully available?

The PLOS Data policy

Reviewer #3: (No Response)

**********

5. Is the manuscript presented in an intelligible fashion and written in standard English?

Reviewer #3: (No Response)

**********

Reviewer #3: (No Response)

**********

what does this mean?). If published, this will include your full peer review and any attached files.

If you choose “no”, your identity will remain anonymous but your review may still be made public.

**Do you want your identity to be public for this peer review?**  For information about this choice, including consent withdrawal, please see our Privacy Policy

Reviewer #3: No

**********

# Editor report: Acceptance letter

**DOI:** 10.1371/journal.pone.0362064.r006

**Authors:** Andrea Cioffi

PONE-D-26-10064R2

PLOS One

Dear Dr. Lindig,

I'm pleased to inform you that your manuscript has been deemed suitable for publication in PLOS One. Congratulations! Your manuscript is now being handed over to our production team.

At this stage, our production department will prepare your paper for publication. This includes ensuring the following:

* All references, tables, and figures are properly cited

* All relevant supporting information is included in the manuscript submission,

* There are no issues that prevent the paper from being properly typeset

You will receive further instructions from the production team, including instructions on how to review your proof when it is ready. Please keep in mind that we are working through a large volume of accepted articles, so please give us a few days to review your paper and let you know the next and final steps.

Lastly, if your institution or institutions have a press office, please let them know about your upcoming paper now to help maximize its impact. If they'll be preparing press materials, please inform our press team within the next 48 hours. Your manuscript will remain under strict press embargo until 2 pm Eastern Time on the date of publication. For more information, please contact onepress@plos.org.

You will receive an invoice from PLOS for your publication fee after your manuscript has reached the completed accept phase. If you receive an email requesting payment before acceptance or for any other service, this may be a phishing scheme. Learn how to identify phishing emails and protect your accounts at https://explore.plos.org/phishing.

If we can help with anything else, please email us at customercare@plos.org.

Thank you for submitting your work to PLOS One and supporting open access.

Kind regards,

PLOS One Editorial Office Staff

on behalf of

Dr. Andrea Cioffi

Academic Editor

PLOS One
